# Meeting Abstracts: London Trauma Conference, London Cardiac Arrest Symposium, London Pre-hospital Care Conference 2018: London, United Kingdom. 10-13 December 2018

**DOI:** 10.1186/s13049-019-0639-x

**Published:** 2019-07-19

## 1) Predictive utility of Delta neutrophil index (DNI) in patients with multiple organ failure (MOF) after severe traumatic injury

### Yong Won Kim^1^, Eun Seok Hong^1^, Woo Jin Jung^2^, Oh Hyun Kim^2^

#### ^1^Department of Emergency Medicine, University of Ulsan College of Medicine, Ulsan University Hospital, Ulsan, Republic of Korea; ^2^Department of Emergency Medicine, Yonsei University Wonju College of Medicine, Wonju, Republic of Korea

##### **Correspondence:** Oh Hyun Kim (ardentem@gmail.com)

**Background**

Post-injury MOF is the result of a systemic uncontrolled inflammatory response and it is the one of leading cause of late post-injury mortality. [1] DNI may serve useful marker for early diagnosis of inflammatory condition. [2] We ascertained whether DNI might be useful in prediction of post-injury MOF.

**Methods**

We performed to retrospective study of a level 1 trauma center database from January 2010 to March 2014. Trauma patients more than 18 years of age with an Injury Severity Score of >15 points and consecutive admission to intensive care unit or operation were included. Organ function was evaluated according to the Sequential Organ Failure Assessment (SOFA) score while DNI were measured repetitively every morning.

**Results**

Overall, 96 patients were enrolled in this study. No differences were found referring to age, gender, laboratory value, injury distribution or overall injury severity between patients with and without MOF. Compared with non-MOF group, the MOF group had a significantly higher amount of fresh frozen plasma transfusion and a higher mortality, statistically. As a result of repeated measurement of DNI values for 14 days, DNI values were significantly higher in MOF group than in non-MOF group, and the trends of DNI changes were similar between two groups. On the fifth day of hospitalization, area under the ROC curve of DNI in MOF group was 0.662 (0.553 to 0.770).

**Conclusions**

DNI values were significantly higher in the MOF patients than in the non-MOF patients following 14 days after severe traumatic injury.

Conflict of interest: All authors have nothing to declare on conflict of interest.

**References**

1. Dewar D, Moore FA, Moore EE, Balogh Z. Postinjury multiple organ failure. Injury. 2009;40(9):912-8.

2. Seok Y, Choi JR, Kim J, Kim YK, Lee J, Song J, et al. Delta neutrophil index: a promising diagnostic and prognostic marker for sepsis. Shock. 2012;37(3):242-6.

## 2) A case series of resuscitative endovascular balloon occlusion of the aorta (REBOA) for noncompressible torso hemorrhage

### Byungchul Yu, Giljae Lee, Jungnam Lee, Daesung Ma

#### Trauma Surgery, Gachon University Gil Medical Center, Incheon, Republic of Korea

##### **Correspondence:** Byungchul Yu (saint7331@gmail.com)

**Background**

Resuscitative endovascular balloon occlusion of the aorta (REBOA) is using as an adjunctive management for profound shock in some trauma centers. We report our early experience of REBOA to describe implementation and possible indications of REBOA.

**Method**

Descriptive case series of REBOA in profound shock due to noncompressible torso hemorrhage (NCTH) at a single Korean trauma center.

**Results**

All cases (n=12) were done for blunt mechanisms. Six cases (50%) were REBOA in zone 1 and six cases (50%) in zone 3. Mean pre-occlusion systolic blood pressure (SBP) was 54.7 and post-occlusion SBP was 95.3. Eleven patients (91.7%) survived at trauma bay and seven (58.3%) survived for 24 hours. Four patients (33.3%) survived and discharged without neurologic deficit and all of them were zone 3 occlusion.

**Conclusion**

REBOA can be effective method of aortic control in profound shock due to NCTH. In our series, especially zone 3 REBOA for pelvic hemorrhage showed better outcomes.

## 3) Serratus anterior plane block in the management of thoracic trauma – a local QIP

### Rory McDonald, Ali Watts, Roger Bloomer

#### Department of anaesthetics, King’s College Hospital, London, UK

##### **Correspondence:** Rory McDonald (rory.mcdonald@nhs.net)

**Background**

Analgesia in thoracic trauma has two major problems: the depressant effects of opiates may worsen pulmonary compromise, and a thoracic epidural is often contraindicated due to co-morbidities or spinal injury. Serratus anterior plane (SAP) block is an opioid-sparing regional anaesthetic that circumvents the risks of neuraxial blockade. We set out to formulate a local protocol for its use as an alternative analgesic in thoracic trauma.

**Methods**

The following data was retrospectively collated from medical records for each patient with thoracic trauma undergoing a SAP block: demographics, co-morbidities (including anticoagulation) and position/severity of injury (including flail component). This data enabled calculation of a standardised rib fracture score (RFS, range 3 [simple analgesics required] to >15 [complex analgesia required]) [1]. The time from admission to administration of regional anaesthesia was also noted. SAP blocks were performed under ultrasound guidance using a single bupivacaine bolus with or without subsequent epidural catheter placement and infusion. A pain score (0 = no pain, 10 = highest pain) was recorded pre- and post-block.

**Results**

Over 12 months 51 patients, with a mean age of 55.7 years (s = 18.5), underwent SAP block. The mean RFS was 7.0 (s = 3.6) and 73% had underlying pulmonary injury on radiological imaging. Furthermore, 33% of patients suffered flails. Interestingly, it took an average of 1 day (IQR = 2) from admission for SAP block placement. There was a significant reduction in median pain score of 1.5 points (*U* = 2648.5, *p* = 0.002) post block. Five patients were discounted due to incomplete pain score recording.

**Discussion**

Our practice demonstrated efficacy in pain reduction post SAP block. Following appropriate training, a trust-wide protocol based on this practice was instigated. We are now assessing uptake of the protocol and for a reduction in time to SAP block post admission.

**Reference**

1. May L, Hillermann C, Patil S. Rib fracture management. BJA Education. 2015; 16:26-32.

## 4) Effectiveness of a primary prevention educational package in improving parental knowledge and behaviour in relation to preventable causes of infant hospital admissions and death

### Jasmine J Parkes^1^, Thomas D Farmer^1^, Jieyun Lee^2^, Philip Hyde1^1^

#### ^1^Faculty of Medicine, University of Southampton, Southampton, UK; ^2^Department of Surgery, Basingstoke and North Hampshire Hospital, Basingstoke, UK

##### **Correspondence:** Jasmine J Parkes (jasmineparkes@doctors.org.uk)

**Background**

Unintentional injuries during childhood have two peaks - infancy and adolescence. Many injuries have modifiable factors.^1^ Unintentional injuries cause physical, emotional and financial burden on the child, family and service providers.^2,3^ The aim of this investigation was to deliver and determine if parents/carers retain information in the Primary Prevention Education Package (PPEP).

**Methods**

120 participants completed questionnaires to assess baseline knowledge. Control group (CG) completed the questionnaire 1-month after ‘Save a Baby’s Life’ (SABL) session. Intervention group (IG) completed the questionnaire 1-month after SABL session with additional PPEP. PPEP is an interactive presentation highlighting potential infant hazards. Excel and SPSS were used to analyse data. Independent *t*-tests and paired *t*-tests were used. A P-value ≤0.005 (95% Cl) deemed the results significant.

**Results**

No significant difference in baseline score between CG and IG, P=0.320 (95%Cl, 2.062 to 6.262).

No significant change in CG score between baseline and 1-month, P= 0.406 (95%Cl, 9.425 to 20.282).

Significant change in IG score between baseline and 1-month, P= 0.000 (95%Cl, 15.742 to 26.058)

Significant difference in 1-month score between CG and IG, P=0.005 (95%Cl, 5.196 to 24.661).

**Conclusion**

Results show no baseline difference between CG and IG. At 1 month, there is a significant difference between the two groups, with IG showing a significant improvement on questionnaire score. This shows intensive teaching combined with PPEP improves parent and carers ability to identify and prevent hazards during infancy.

**Acknowledgement**

Project Supervisor - Dr Philip Hyde, consultant paediatric intensivist

Peer Review – Dr John Pappachan, consultant paediatric intensivist

University of Southampton Research and Development Team

Save a Baby’s Life volunteers and attendees

**Trial registration**

University ethics ERGO (14653), NHS ethics NRES (15/LO/2218), and local R&D (RHM CHI0791) were obtained for this project.

**References**

1 Sidebotham P, Fraser J, Fleming P, Ward-Platt M, Hain R. Patterns of child death in UK. Lancet 2014;384: 904-14.

2 Towner E, Dowswell T, Mackereth C, Jarivs S. What works in preventing unintentional injuries in children and young adolescents? An updated systemic review. NHS. Report 22001.

3 Department of Education. DFE-00130-2015. Working together to safeguard children. London: HM Government; 2015, 2015.

## 5) Ionised calcium levels in civilian patients with suspected traumatic haemorrhage following the addition of Calcium Chloride to a pre-hospital transfusion protocol

### Alina Humdani^1^, Anthony Hudson^2,3^, Joanne Griggs^2^, Richard Lyon^2^

#### ^1^ St George’s University of London, Cranmer Terrace, Tooting, London, SW17 0RE; ^2^ Kent, Surrey and Sussex Air Ambulance Trust, Redhill Aerodrome, Redhill, Surrey, RH1 5YP; ^3^ St George's University Hospitals NHS Foundation Trust, Blackshaw Road, London, SW17 0QT

##### **Correspondence:** Alina Humdani (m1500193@sgul.ac.uk)

**Background**

Hypocalcaemia is evident in major trauma patients, compounded by blood product administration, potentially resulting in coagulopathy and cardiac dysfunction. The aims of this study were to ascertain the incidence of hypocalcaemia in civilian trauma patients, and establish whether the introduction of pre-hospital calcium chloride into a transfusion protocol effects admission ionised calcium. Further, establishing clinician adherence to transfusion protocol.

**Methods**

A retrospective observational study of patients declared ‘Code Red’ by Air Ambulance Kent, Surrey & Sussex (AAKSS), between 11 July, 2016 and 25 May, 2018. AAKSS protocol, states that 10mL of intravenous Calcium Chloride (10%) should be administered immediately after the transfusion of 2 units of PRBC (packed red blood cells). Data collected included the total number of units of PRBCs transfused and admission ionised calcium. Results were compared to a historic group. Patient demographics, Injury Severity Score, mechanism of trauma and time of 999 call to in-hospital blood gas were analysed.

**Results**

A total of 153 Code Red activations were identified, data for 71 patients was available for further analysis, and a total of 12 cases were excluded. Therefore, 19 patients were included in the post-calcium group and 40 in the pre-calcium group. 21.1% (*n*= 4) remained hypocalcaemic in the treatment group, compared to 12.5% (*n*= 5) in the non-treatment group (*p*= 0.999). The range of iCa values was similar in both groups.

**Conclusion**

Pre-hospital intravenous Calcium Chloride has been successfully introduced into the transfusion protocol for a Helicopter Emergency Medical Service. Limited by study period, a small sample renders its effect on ionised calcium in civilian patients inconclusive, when compared to a recent military paper [1]. Further analysis by means of a prospective multicentre randomised controlled trial may be indicated.

**Reference**

[1] 1 Kyle, T, Greaves, I, Beynon, A *et al.* Ionised calcium levels in major trauma patients who received blood en route to a military medical treatment facility. Emergency Medical Journal. 2017 November 24 [cited 2018 May 25]. 35(3)176-179. Available from: http;//dx.doi.org/10.1136/emermed-2017-206717

## 6) Point-of-care analysis from intraosseous access within pre-hospital emergency patients

### Milla Jousi^1^, Jouni Nurmi^2^

#### ^1^ FinnHEMS Research and Development Unit, Vantaa, Finland; ^2^ Emergency Medicine, University of Helsinki and Department of Emergency Medicine and Services, Helsinki University Hospital, Helsinki, Finland

##### **Correspondence:** Milla Jousi (milla.jousi@hus.fi)

**Background**

If difficulties with vascular access are encountered, intraosseous (IO) access is used alternatively. Blood aspirated to confirm the correct IO needle position could readily be available for point-of-care (POC) analysis if arterial or venous blood is not available. Analyses of blood gases, acid-base balance, electrolytes, lactate or haemoglobin can provide important information for decision-making. Currently, very little evidence exists about the agreement of IO values with arterial or venous values, especially among haemodynamically unstable patients. [1] The aim of this study was to evaluate the agreement between IO and arterial samples of critically ill pre-hospital patients.

**Materials and methods**

We performed a prospective observational study in a physician staffed helicopter emergency medical (HEMS) unit. We included 14 adult patients who needed an IO access and POC laboratory analysis. We took samples from IO access and artery within 10 minutes of time frame. We analysed the samples with an iSTAT® POC analyser and compared the results using Bland-Altman statistical method. The patients suffered from brain injury or suspected intracranial hemorrage (n=4), intoxication (n=3), severe trauma (n=3), cardiac arrest (post-resuscitation) (n=3) or ketoacidosis (n=1).

**Results**

Agreement between IO and arterial samples was good for sodium (bias -2.5 mmol/l, 95% CI 3.8 - -8.8), pH (bias -0.02, 95% CI 0.07 - -0.11) and base excess (bias 1.2 mmol/l, 95% CI 5.3 - -2.8). IO analysis of haemoglobin proved not to be reliable because of large variance of the bias (bias -0.6 g/l, 95% CI 89.2 - -90.3). Analyses of potassium from IO access showed a bias of 2.0 mmol/l (95% CI 3.72 – 0.33).

**Conclusions**

Fairly good agreement between IO and arterial samples exists for sodium, base excess and pH. Analysis of IO haemoglobin is not reliable. Potassium values are on average 2.0 mmol/l higher in IO samples than in arterial blood.

**Reference**

1. Jousi M, Laukkanen-Nevala P, Nurmi J. Analysing blood from intraossseous access: a systematic review. Eur J Emerg Med. 2018 Aug 17. [Epub ahead of print]

## 7) Trauma induced Acute Kidney Injury

### Zane Perkins^1^, Gabriella Captur^2^, Ruth Bird^3^, Liam Gleeson^4^, Ben Singer^5^, Benjamin O'Brien^5^

#### ^1^Trauma Surgeon Royal London Hospital. UK; ^2^Cardiology SPR Barts Heart. London UK; ^3^Anaesthetics SPR Royal London Hospital. UK; ^4^ICU SHO Barts Heart. London UK; ^5^Anaesthetics Consultant Barts Heart. London UK

##### **Correspondence:** Ruth Bird (RuthHbird@doctors.org.uk)

**Introduction**

Injured patients are at risk of developing acute kidney injury (AKI), which is associated with increased morbidity and mortality. The aim of this study was to describe the incidence, timing, and severity of AKI in a large trauma population, identify risk factors for AKI, and report mortality outcomes. [1-3]

**Methods**

A prospective observational study of injured adults, who met local criteria for trauma team activation, and were admitted to a UK Major Trauma Centre. AKI was defined by the Kidney Disease Improving Global Outcomes (KDIGO) criteria. Multivariable logistic regression and Cox proportional hazard modelling was used to analyse parameters associated with AKI and mortality.

**Results**

Of the 1410 patients enrolled in the study, 178 (12.6%) developed AKI. Age; injury severity score (ISS); admission systolic blood pressure, lactate and serum creatinine; blood transfusion requirements in first 24 hours and administration of nephrotoxic therapy were identified as independent risk factors for the development of AKI. Patients that developed AKI had significantly higher mortality in the first 65 days post trauma, than those with normal renal function (47/178 (26.4%) versus 128/1232 (10.4%); RR 1.22 (1.12 to 1.34); p<0.0001). After adjusting for other clinically prognostic factors, AKI was an independent risk factor for mortality.

**Conclusions**

AKI is a frequent complication following trauma and is associated with prolonged hospital length of stay and increased mortality. Future research is needed to improve our ability to rapidly identify those at risk of AKI, and develop resuscitation strategies that preserve renal function in trauma patients.

**References**

1) Lozano R, Naghavi M, Foreman K, et al. Global and regional mortality from 235 causes of death for 20 age groups in 1990 and 2010: a systematic analysis for the Global Burden of Disease Study 2010. Lancet. 2012; 380:2095-2128.

2) Murray CJ, Vos T, Lozano R, et al: Disability-adjusted life years (DALYs) for 291 diseases and injuries in 21 regions, 1990-2010: a systematic analysis for the Global Burden of Disease Study 2010. Lancet. 2012; 380:2197-2223.

3) Vos T, Flaxman AD, Naghavi M, et al: Years lived with disability (YLDs) for 1160 sequelae of 289 diseases and injuries 1990-2010: a systematic analysis for the Global Burden of Disease Study 2010. Lancet. 2012; 380:2163-2196.

## 8) Reducing door to cath lab time – the introduction of a team based Out of Hospital Cardiac Arrest protocol

### Matt Govier^1^, Ivan Collin^2^, Matt Thomas^3^

#### ^1^ ST5 Anaesthetics / ICM – Bristol School of Anaesthesia, UK; ^2^ ST5 Acute Medicine / ICM – Bristol School of Anaesthesia, UK; ^3^ Consultant Intensivist, University Hospitals Bristol, Bristol, UK

##### **Correspondence:** Matt Govier (mattgovier@doctors.net.uk)

**Background**

The Bristol Heart Institute receives 300 Out of Hospital Cardiac Arrest (OHCA) patients per annum. There was recognition of the need to streamline the initial review, treatment, transport and safe disposal of this patient cohort. Prior to intervention, the OHCA patient was initially managed predominantly by Emergency Department (ED) staff, with ad hoc requests for input from Anaesthetics, ICU, Cardiology and Radiology. In previous local evaluations of this service model, there was considerable inter-patient variation and unnecessary delays in definitive treatment.

**Methods**

We sought to improve this situation by introduction of an OHCA team, who would be present in ED from the time of patient arrival. The OHCA team were summoned to ED by means of a pre-alert bleep. The team comprised of senior decision makers from relevant specialties, thus allowing rapid intervention and onward disposal.

**Results**

We collected data following the introduction of the team and demonstrated a reduction in door to cath lab procedure start time (DtP). The mean DtP post team introduction was 50 minutes, compared with 72 minutes the previous year. We also noted a relatively high palliation rate in ED. On notes review this appeared to be appropriate and due to the rapid input of multiple specialties present at the bedside.

We also sought to evaluate where further efficiency savings could be made. This highlighted an excess time spent in ED. The reasons were; poor preparation, excess time on unnecessary procedures and non-time critical investigations.

**Conclusion**

The introduction of an OHCA team has improved time factors and consistency in our institution. Future work is focused on:

- Introducing a standard resus bay set up, including pre-prepared ventilators, arterial line sets and inotropic infusions;
- Instigating a single MDT combined proforma and protocol;
- Providing in-situ simulation training for the OHCA team.

## 9) An audit of time to surgery for distal radius fractures requiring surgery at two district general hospitals near Sheffield - is compliance with the NICE, BOA and BSSH guidelines feasible?

### Henry O’Brien^1^, Armughan Azhar^1^, John Wright^1^, Abraham Odumala^2^, Lawrence Majkowski^2^

#### ^1^Chesterfield Royal Hospital, Chesterfield, S44 5BL; ^2^Barnsley Hospital, Barnsley, S75 2EP

##### **Correspondence:** Henry O’Brien (Henry.o’brien1@nhs.net)

**Background**

NICE, the British Orthopaedic Association (BOA) and the BSSH (British society for surgery of the hand) provide guidance for the timing of surgery for distal radius fractures (DRFs) requiring surgery. They all agree that for intra-articular DRFs surgery “should be performed within 72 hours of injury and within one week for extra articular fractures.”^1^ Our aim was to audit the compliance to this guideline.

**Methods**

The same time period, January 1^st^ to April 30^th^ 2017, at the two different hospitals was used. Trauma lists for both hospitals were reviewed and any adult patient with a distal radius fracture undergoing surgery was identified. Radiographs and case notes were reviewed retrospectively for the type of fracture, time and date of presentation and injury, when the decision to operate was made, age of the patient at surgery and the time and date of surgery.

**Results**

53 patients were audited in total. At Chesterfield Royal Hospital (CRH) 29 patients were audited with an overall compliance of 72% for all DRFs requiring surgery whereas at Barnsley Hospital (BH) this was 58% for the 24 patients audited.

69% of intra-articular DRFs had surgery within the guideline target at CRH and this increased to 75% for extra-articular DRFs. 44% of intra-articular DRFs had surgery within the guideline target at BH and this increased to 67% for extra-articular DRFs.

At BH the mean age at time of surgery was 56 (age range 26 to 84) and at CRH this was 55 (age range 27 to 84).

**Conclusions**

This highlights that the time to surgery for DRFs is not being met. Adherence to the guidelines could be improved and perhaps a national collaborative approach could improve this. Although the acceptable delay for DRFs requiring surgery is debatable in itself. Our audit recommendations will be re-audited locally in 2019.

**Reference**

1. Best practice for management of Distal Radial Fractures (DRFs). Published by British Orthopaedic Association and British Society for Surgery of the Hand 2018

## 10) Survey of long-distance visitors to intensive care units at a regional major trauma centre in the United Kingdom

### Stefan T Kulnik^1^, Anna-Karynka Wulf^2^, Christopher Brunker^3^

#### ^1^Faculty of Health, Social Care and Education, Kingston University and St George’s, University of London, London, UK; ^2^Neuro-Therapies Department, St George’s University Hospitals NHS Foundation Trust, London, UK; ^3^Neuro Intensive Care Unit, St George’s University Hospitals NHS Foundation Trust, London, UK

##### **Correspondence:** Stefan T Kulnik (s.t.kulnik@sgul.kingston.ac.uk)

**Background**

Little attention has been paid to the experiences of long-distance visitors at major trauma centres (MTCs). This study aimed to investigate experiences of long-distance visitors of major trauma patients admitted at St George’s Hospital, South London, to identify needs and opportunities for service improvement.

**Method**

An anonymous postal survey was conducted. A newly developed questionnaire was posted to visitors of major trauma patients, who had been admitted to intensive care at the study site between January 2016 and July 2018. Included were next of kin with ordinary residence further than one hour’s drive from the MTC (estimated according to Google Maps). Visitors were excluded, if the patient had been admitted for under 2 days.

**Results**

Forty-seven out of 103 visitors responded (response rate 45.6%). Median (range) driving distance between respondents’ residence and the MTC was 57.8 km (28.8-331.5). Median (range) number of days respondents visited at the MTC was 18 (1-200). Relative to patients’ length of stay, respondents visited on 16.7% to 100% of days, with median 97.4% and lower quartile 70.7%. Mean (SD) duration of visitors’ one-way journey to the MTC was 103 minutes (45), and mean (SD) cost of one return journey was £22.4 (16.2). Visitors rated the staff and quality of care at the MTC highly. They also described their often challenging circumstances, negotiating the physical, psychological, emotional and financial impact of the situation. Several areas for service improvement were highlighted. These included car parking, signposting on and around the MTC site, information provision, waiting areas, and accommodation at or nearby the MTC.

**Conclusion**

This was the first study to address the issue of long-distance visiting in major trauma care in the United Kingdom. The study has described the experiences of visitors, and locally identified opportunities for ameliorating visitors’ stress points during this difficult time.

## 11) Thames Valley Air Ambulance Cardiac Arrest Study: What is the value added of an advanced critical care team during an out-of-hospital cardiac arrest?

### James Raitt^1^, Stewart McMorran^1^, Richard Company^1^, Joseph Fennelly^2^

#### ^1^Thames Valley Air Ambulance Service, Oxfordshire, UK; ^2^ Stoke Mandeville Hospital, Buckinghamshire, UK

##### **Correspondence:** Joseph Fennelly (jfennelly@doctors.org.uk)

**Background**

Cardiac arrests form a significant part of the workload of Thames Valley Air Ambulance (TVAA). There have been several studies to identify variables associated with higher incidence of patient survival to discharge that could be used to influence clinical decision-making [1, 2]. Aim: To determine the mechanism, response, interventions and outcomes of out-of-hospital cardiac arrests (OHCA) attended by TVAA.

**Methods**

This was a six-month service evaluation and prospective study. Subjects included all patients attended by a TVAA asset (helicopter or emergency response vehicle (ERV)) who had an OHCA from November 2017 to May 2018.

**Results**

91 patients (mean age of 58) were included in the study, 9 of which were traumatic OHCA. 52% were attended to by helicopter and 43% by the ERV. 57% of patients were declared dead at the scene and a total of 31 patients were transported to hospital of which six survived to discharge. Interventions performed by TVAA included: LUCAS (64% of patients); echocardiography (34%); and Pre Hospital Emergency Anaesthesia (PHEA) (24%).

**Discussion**

Our results showed similar survival to discharge for OHCA in England as outlined in current literature [2]. However due to a greater proportion of TVAA patients being declared dead at scene, we found a smaller number of our patients to be declared dead in the Emergency Department (20%) compared to the national figure (42%) [2, 3]. This may represent that early identification on whether further resuscitation is futile can reduce the workload on receiving Emergency Departments. Our study also demonstrated that TVAA provided a number of interventions that are not available from a standard South Central Ambulance Service crew (LUCAS, echocardiography, PHEA, thoracostomies and drugs administration beyond the ALS algorithm). These early interventions provide potentially life-saving treatments and also aid for TVAA Clinicians to make informed decisions regarding resuscitation attempts.

**Acknowledgements**

Thank you to the support from Thames Valley Air Ambulance crew in the collection and upload of the data used in this study

**References**

1. Perkins G, Jacobs I, Nadkarni V, Berg R, Bhanji F, Biarent D et al. Cardiac Arrest and Cardiopulmonary Resuscitation Outcome Reports: Update of the Utstein Resuscitation Registry Templates for Out-of-Hospital Cardiac Arrest. Resuscitation. 2015;96:328-340.

2. Statistics - Ambulance Quality Indicators [Internet]. England.nhs.uk. 2018 [cited 12 July 2018]. Available from: https://www.england.nhs.uk/statistics/statistical-work-areas/ambulance-quality-indicators/

3. Perkins G, Lall R, Quinn T, Deakin C, Cooke M, Horton J et al. Mechanical versus manual chest compression for out-of-hospital cardiac arrest (PARAMEDIC): a pragmatic, cluster randomised controlled trial. The Lancet. 2015;385(9972):947-955.

## 12) Intraosseous access for trauma patients in the era of damage control resuscitation: A literature review

### Joseph A Tyler^1^, Henry D De’Ath^2^

#### ^1^Department of Anaesthesia, Queen Elizabeth Hospital, Birmingham, UK; ^2^ Centre for Trauma Sciences, Queen Mary University, London, UK

##### **Correspondence:** Joseph A Tyler (joseph.tyler@nhs.net)

**Background**

Damage Control Resuscitation (DCR) has become a standard of care for the seriously injured [1,2]. Intraosseous catheters continue to be recommended in trauma resuscitation. Their utility has recently been debated due to concerns regarding inadequate flow rates during blood transfusion and the potential for haemolysis [3]. This review summarises the evidence for intraosseous catheters in trauma in the modern era of DCR and highlights areas for future research.

**Methods**

A PubMed and Embase search for articles published from January 1990 to August 2018 using the terms (“intra-osseous access” OR “intraosseous access” OR “IO access”) AND trauma. All manuscripts describing the use of an intraosseous catheter in the resuscitation of one or more trauma patients were eligible. Studies including a mixture of trauma and non-trauma patients were excluded if they did not differentiate between the groups in their results section. Animal studies, cadaveric studies and those involving healthy volunteers were also excluded.

**Results**

There were nine eligible studies, comprising of 1218 trauma patients and 1430 individual device insertions. The overall insertion success rate was 95.3% and the incidence of complications 1%. Flow-rate data was extremely scarce, being available for only two transfusions of packed red cells in a single patient. Haemolysis was poorly reported.

**Conclusion**

Intraosseous catheters have high insertion success rates and a low incidence of complications in trauma patients. There is a remarkable paucity of objective flow rate data for blood transfusion via intraosseous catheters in this population, although much anecdotal evidence advocating their use exists. Randomised controlled trials comparing intraosseous versus peripheral and central venous access in this setting are absent but required, as are studies providing flow rate data. Existing evidence suggests that intraosseous transfusion is not associated with haemolysis, however further studies in humans are necessary.

**References**

1. Rossaint R, Bouillon B, Cerny V, Coats T, Duranteau J, Fernandez-Mondejar E et al. The European guideline on management of major bleeding and coagulopathy following trauma: fourth edition Crit Care. 2016; 20: 100

2. National Institute for Health and Care Excellence (NICE). Major trauma: assessment and initial management: NICE Guideline [NG39]. 2016. Available from: https://www.nice.org.uk/guidance/ng39

3. Harris M, Balog R, Devries G. What is the evidence of utility for intraosseous blood transfusion in damage-control resuscitation? *J Trauma Acute Care Surg.* 2013; 75: 904-6

## 13) Weight of Splints and Casts used in Conservative Treatment of Distal Radius Fractures

### Nienke de Laat, Lysanne van Silfhout, Albert F Pull ter Gunne, Edo J Hekma

#### Department of surgery, Rijnstate, Arnhem, the Netherlands

##### **Correspondence:** Lysanne van Silfhout (LvanSilfhout@Rijnstate.nl)

**Background**

Distal radius fractures are the most common fracture in the general population, accounting for 25% of fractures in children and up to 18% in adults aged 65 years and older.[1] It is treated conservatively with cast immobilization in approximately 70%.[2] This study aimed to explore the diversity in quality of casts used in conservative treatment of distal radius fractures.

**Methods**

Patients with a distal radius fracture who presented for changing or removing the cast at the Rijnstate hospital in Arnhem, the Netherlands, were included in this study. Exclusion criteria were open fractures, fractures requiring surgery, age below 18 years, inability to cooperate and casts applied in other hospitals. Dependent variables were weight and length of the cast. Independent variables were age, BMI and length of the arm. The length of the forearm was obtained through a 3D scan after removal of the cast. Differences between casts applied by plaster technicians and emergency physicians were also assessed.

**Results**

In total 29 patients were included. Three groups, based on casting material and technique, were defined: mineral splint (n=8) and circular plaster cast (n=21). The average weight was respectively 290.6 ± 66.2 and 99.9 ± 8.0.

**Conclusion**

On average, mineral splints applied by emergency physicians weigh more than plaster casts applied by plaster technicians; respectively 291 ± 66 gram and 100 ± 8 gram. The weight and length of mineral splints correlates moderately with the length of the arm. The weight and length of the circular plaster casts, applied by plaster technicians, shows minimal correlation with the arm length.

**References**

1. Nellans KW, Kowalski E, Chung KC. The Epidemiology of Distal Radius Fractures. Hand Clin. 2012; 28(2):113–125.

2. Chung KC, Spilson SV. The frequency and epidemiology of hand and forearm fractures in the United States. J. Hand Surg. Am. 2001; 26(5):908–915.

## 14) Time to definitive care in English major trauma networks

### Nicholas R Haslam^1^, Omar Bouamra^2^, Chris Moran^3^, David J Lockey^4^

#### ^1^ Barts & The London School of Anaesthesia, Barts NHS Trust, London, UK; ^2^ Trauma Research and Audit Network, University of Manchester, Salford, UK; ^3^ Orthopaedic and Trauma Surgery, Queens Medical Centre, Nottingham, UK; ^4^ Centre for Trauma Sciences, Blizard Institute Queen Mary University, London, UK

##### **Correspondence:** Nicholas R Haslam (nicholashaslam@nhs.net)

**Background**

Significant mortality improvements have been demonstrated following implementation of English regional trauma networks in 2012.[1] Timely transfer of seriously injured patients to definitive care is a key indicator of network performance. This study describes timelines from emergency service (EMS) activation to definitive care arrival and key interventions for severely injured patients between 2013 and 2016.

**Methods**

Data was collected from the Trauma Audit and Research Network on patients with injury severity scores above 15. Outcomes included time from EMS activation to arrival at a Trauma Unit (TU) or Major Trauma Centre (MTC); to computerised tomography (CT); to urgent surgery; and mortality.

**Results**

Compared to direct transfer, secondary transfer was associated with increased time to CT [419 vs 120 minutes (p < 0.0001)]; increased time to urgent surgery [434 vs 262 minutes (p < 0.0001)]; and increased mortality [19.6(CI95%:16.9-22.3) vs 15.7(CI95%:14.7-16.7)].

Time to CT was longer at TUs compared to MTCs [189 vs 120 minutes (p < 0.0001)] as was time to urgent surgery [322 vs 262 minutes (p < 0.0001)]. Transfer time and time to CT increased from 2013-2016 (p < 0.0005). There was significant variation between regional networks for transfer time; time to CT; and time to urgent surgery (p < 0.0001).

**Conclusion**

Patients with serious injuries who underwent secondary transfer had significantly delayed imaging, surgery and increased mortality. CT and urgent surgery were performed more quickly in MTCs than TUs. This may suggest that where safe to bypass these interventions are performed more quickly by going directly to an MTC. Our data suggests that key timelines failed to improve since launch of trauma networks and that significant regional variation in timelines remains. This may indicate that there is still scope for improvement in the early identification, transfer and treatment of severely injured patients in UK practice.

**Reference**

1. Moran CG, Lecky F, Bouamra O, Lawrence T, Edwards A, Woodford M, et al. Changing the System - Major Trauma Patients and Their Outcomes in the NHS (England) 2008–17. EClinicalMedicine. 2018 Aug 1;2–3:13–21.

## 15) Acute Stroke Treatment by a Physician-based Emergency Services Team in a Mobile Stroke Unit

### Karianne Larsen^1^, Lars H Tveit^2^, Henriette S Jæger^1^, Maren R Hov^1^, Jo Røislien^1^, Volker Solyga^2^, Christian G Lund^3^, Kristi G Bache^1^

#### ^1^The Norwegian Air Ambulance Foundation, Oslo, Norway; ^2^Department of Neurology, Østfold Hospital, Kalnes, Norway ; ^3^Department of Neurology, Oslo University Hospital, Oslo, Norway

##### **Correspondence:** Karianne Larsen (karianne.larsen@norskluftambulanse.no)

**Background**

Thrombolytic therapy in acute ischemic stroke is more effective when given early after symptom onset, [1] and different models of Mobile Stroke Units (MSUs) show a reduction in time to treatment in ischemic stroke [2]. In Treat-NASPP (Norwegian Acute Stroke Prehospital Project) we investigate if a non-neurologist physician-based emergency medical services (EMS) team is timesaving and safe in an MSU-system.

**Method**

Treat-NASPP is an ongoing, prospective, controlled intervention study. Patients with onset of stroke symptoms within the last 4 hours are included. The MSU-team mimics the Norwegian Helicopter Emergency Medical Services (HEMS) crew, and consists of an anesthesiologist, a nurse and a paramedic. The control group is the ordinary pathway.

**Results**

Median (quartiles) time from symptom onset to thrombolytic therapy is 92 minutes (64-165) in the MSU compared to 121 (100-162) in the control group, p=0,102. Median (quartiles) time from dispatch to thrombolytic therapy is 36 minutes shorter in the MSU, 54 (48-63) vs 90 (71-96), p=0.003. Median (quartiles) modified Rankin Scale day 90 is 0 (0-1) in the MSU-group and 0 (0-1,75) in the control. No serious adverse events or symptomatic intracranial hemorrhages have occurred.

**Conclusion**

Preliminary results indicate that a physician-based EMS crew in a mobile stroke unit, is timesaving and safe.

**References**

1. Lees KR, Bluhmki E, von Kummer R, Brott TG, Toni D, Grotta JC, et al. Time to treatment with intravenous alteplase and outcome in stroke: an updated pooled analysis of ECASS, ATLANTIS, NINDS, and EPITHET trials. Lancet. 2010;375(9727):1695-703.

2. Fassbender K, Grotta JC, Walter S, Grunwald IQ, Ragoschke-Schumm A, Saver JL. Mobile stroke units for prehospital thrombolysis, triage, and beyond: benefits and challenges. Lancet Neurol. 2017;16(3):227-37.

## 16) The experience of Community First Responders (CFRs) who respond to Out-of-Hospital Cardiac Arrest in Ireland

### Tomas Barry^1^, Suzanne Guerin^2^, Gerard Bury^1^

#### ^1^Centre for Emergency Medical Science, School of Medicine, University College Dublin. Ireland; ^2^School of Psychology, University College Dublin. Ireland

##### **Correspondence:** Tomas Barry (tomas.barry@ucd.ie)

**Background**

Successful resuscitation from out-of-hospital cardiac arrest (OHCA) is largely determined by the early availability of CPR and defibrillation within the community. Ireland has a heterogeneous settlement pattern with many rural communities being situated remote from ambulance service resources. In many instances a timely response to OHCA can only be achieved if it comes from within the community where cardiac arrest occurs.

Over the past ten years in Ireland volunteer community first responder (CFR) schemes have been developed. These schemes involve ‘lay’ volunteers that are present in the community, trained in basic life support and equipped with a defibrillator. CFRs are alerted in real time to OHCA via text message from the ambulance service.

**Methods**

A qualitative research study was undertaken to provide an in-depth understanding of CFRs motivation to participate and the issues they encounter when responding to OHCA. In-depth, semi-structured interviews were carried out with twelve CFRs operating from nine geographically diverse CFR schemes. Interviews were recorded and transcribed. Thematic analysis [1] was undertaken. NVIVO software was utilised.

**Results**

CFRs were motivated by a variety of altruistic, personal and community factors.

CFR care has evolved beyond BLS care. It is a sophisticated entity that includes complex decision making, a significant component dedicated to the provision of psychosocial support for bystanders and family members and also team working with the ambulance service staff during ongoing resuscitation.

**Conclusion**

A range of issues including appropriate recruitment, formal health systems supports, holistic training and volunteer wellbeing need to be considered where health systems deploy CFRs to provide cardiac arrest care.

**Reference**

1) Braun V & Clarke V. Using thematic analysis in psychology. Qualitative Research in Psychology. 2006; 3(2):77-101.

## 17) Massive transfusion protocol (MTP) started by pre-hospital equipe: an Italian experience

### Simone Celi^1^, Marco Botteri^1^, Alberto Piacentini^2^, Giovanna Perone^1^

#### ^1^AAT 118, ASST degli Spedali Civili di Brescia, Brescia, Italy; ^2^Cardio-thoracic ICU, Jilin Heart Hospital, Changchun, China

##### **Correspondence:** Simone Celi (s.celi@areu.lombardia.it)

**Background**

Severe trauma is one of leading causes of death and injury in Europe.

Administration of tranexamic acid (TXA), and blood products, in life-threatening traumatic shock has been already proven to be beneficial. [1, 2]

At our institution a massive transfusion protocol (MTP) has been developed. On scene triggering criteria are: immediate administration of one TXA gram intravenously on scene, and alerting destination hospital for immediate availability need of ready blood products at arrival.

**Methods**

Revision of all MTP activations over a 6 years period (October 2012 - July 2018).

Criteria triggering MTP protocol included: SBP < 90 mmHg unresponsive to fluid challenge, HR >110 bpm (3) non controllable active bleeding (4) penetrating, not compressible, trauma (5) 3^th^/4^th^ ATLS class hemorrhagic shock.

All patients candidate to MTP were reviewed. We analyzed mechanism of injury (MOI), age, sex, SBP, HR, Hemoglobin, total amount, and type of blood products.

**Results**

162 MTP total candidates, administration of TXA resulted in 68 patients (42%), 125 patients received blood products (77.1%).

MOI: : Motor Vehicle Crash (MVC) 61,8%, fall from height 13,7%, penetrating trauma 4,4%, work related accidents (3,8%), unknown mechanism 10%, medical conditions 2,4% and “other” 3,8%.

Mean patient age 46 years (range 15-90). MTP erroneously activated in 4 patients due to medical causes.

Mortality and morbidity: Patients survived to hospital discharge 85,4%, declared dead on scene (1,9%), died in A&E 6,3%, in ICU (5,7%) and in O.T. (0,7%).

Mean SBP was 96 mmHg (range 40-150), mean HR 107 bpm (range 32-180).

Observed transfusion data:

RBC (879) → 7.14 x patient

FFP (676) → 6.25 x patient

PLT (61) → 1.96 x patient

**Conclusion**

Development of simple protocol allowed immediate identification of severely traumatized patients who required immediate resuscitation with blood products directly in emergency department.

**References**

1**.**Shakur H,Roberts I,et al. Effects of tranexamic acid on death, vascular occlusive events, and blood transfusion in trauma patients with significant haemorrhage (CRASH-2): a randomized, placebo-controlled trial. Lancet. 2010;376(9734):23-32

2.Weaver A.E.,Hunter-Dunn C.,et al. The effectiveness of a “Code red” transfusion request policy initiated by pre-hospital physician. Injury,2016,47(1):3-6

## 18) Resuscitative endovascular balloon occlusion of the aorta: A single trauma center experience in Korea

### Dong Hun Kim^1^, Seok Won Lee^1^, Ye Rim Chang^1^, Jeongseok Yun^1^, Seokho Choi^1^, Sung Wook Chang^2^, Jung-Ho Yun^3^

#### ^1^Department of Trauma Surgery, Trauma Center, Dankook University Hospital, Cheonan, Republic of Korea; ^2^Department of Thoracic and Cardiovascular Surgery, Trauma Center, Dankook University Hospital, Cheonan, Republic of Korea; ^3^Department of Neurosurgery, Trauma Center, Dankook University Hospital, Cheonan, Republic of Korea

##### **Correspondence:** Sung Wook Chang (changsw3@naver.com)

**Background**

Resuscitative endovascular balloon occlusion of the aorta (REBOA) as minimally invasive alternative to open aortic cross clamping to provide temporary aortic occlusion can be a bridging modality for damage control resuscitation [1,2]. We present experiences of REBOA in patients with noncompressible torso hemorrhage after multiple blunt trauma in Korea.

**Methods**

A prospective data of trauma patients undergoing REBOA at a level I trauma center from 2016 to 2018 was analyzed retrospectively. The inclusion criteria were blunt trauma patients with abdominopelvic exsanguination and hypotensive hemodynamics.

**Results**

Of 31 blunt trauma patients underwent REBOA, 25 who had abdominopelvic hemorrhage were eligible for the selection criteria. The median age of REBOA patients was 52 years (interquartile range, 44-69), and 72% were male. The median injury severity score was 32 (25-43). Of them, aorta was occluded in zone I of 17 patients (68%) with abdominal organ injuries and zone III of 8 (32%) with unstable pelvic fracture. Seven patients (28%) survived with damage control procedure following REBOA (6 zone I, 1 zone III). The median systolic blood pressure (SBP) of 99 mmHg (87-110) after REBOA was significantly higher than SBP of 55 mmHg (47-60) before (p < 0.001). In the survivor group of REBOA, as compared with the nonsurvivor, there were significant higher rates of hemorrhage of a single abdominal organ (85.7% vs. 11.1%, p =0.001), high Glasgow Coma Scale (15 [9-15] vs. 7 [3-12], p = 0.011), low requirement of RBC transfusion (15 [11-19] vs, 36 [18-50] units, p = 0.001), and short aortic occlusion time (84 [42-122] vs. 154 [ 55-253] minutes, p = 0.049).

**Conclusion**

REBOA might be a favorable resuscitative modality, especially for traumatic exsanguination of a solitary abdominal organ. Systemic treatment strategies for definitive bleeding control of severe multiple abdominopelvic injuries would be needed for survival following REBOA.

**References**

1. Moore LJ, Martin CD, Harvin JA, et al. Resuscitative endovascular balloon occlusion of the aorta for control of noncompressible truncal hemorrhage in the abdomen and pelvis. Am J Surg. 2016;212(6):1222-1230.

2. Abe T, Uchida M, Nagata I, et al. Resuscitative endovascular balloon occlusion of the aorta versus aortic cross clamping among patients with critical trauma: a nationwide cohort study in Japan. Crit Care. 2016;20(1):400.

## 19) Using key performance indicators to drive quality improvement in pre-hospital blood transfusion: The Thames Valley Air Ambulance Approach

### James Raitt^1^, Pip Lewis^2^, James Dearman^1^

#### ^1^Thames Valley Air Ambulance, RAF Benson, Oxford, UK; ^2^Emergency Department, St Mary’s Hospital, Imperial College NHS Healthcare Trust, London, UK

##### **Correspondence:** Pip Lewis (pip.lewis1@nhs.net)

**Background**

Pre-hospital blood transfusion improves survival and outcomes from major trauma. Thames Valley Air Ambulance (TVAA) has been carrying blood products since 2013. The service has recently introduced Key Performance Indicators (KPIs) to audit its use of pre-hospital blood transfusion.

**Methods**

KPIs were selected after consulting an expert panel of TVAA clinicians and literature search for current papers concerning the use of KPIs in a pre-hospital environment. Seven auditable domains; rationale for transfusion, compliance with standard operating procedures (SOPs), hypothermia management, tranexamic acid (TXA) administration, evidence of bleeding in hospital and complications from transfusion, have been implemented since December 2017 for prospective analysis of current pre-hospital blood transfusion practice. Decision whether the use of blood was justified was determined by peer review.

**Results**

In an 8-month period (Nov 2017-June 2018) there were 23 pre-hospital blood transfusions. One case was missing all data therefore 22 cases were analysed. One case was obstetric, all others were trauma. Mortality was 22.7%. A total of 38 units of RBC and 38 units of FFP were given. Domains with the highest percentage of achievement were: Rationale for transfusion in line with blood transfusion SOP (100%); No blood transfusion complications (100%) and overall was the use of blood justified (100%).

**Conclusion**

The use of KPIs demonstrated areas of good quality practice and led to improvements in equipment, processes and documentation. Compliance was lower with aggressive management of hypothermia and TXA delivery. An element of this was due to poor recording, which will be addressed by moving to an alternative patient record system. A previous review of 2 years TVAA RBC use suggested blood was on occasion given outside of the SOP, the introduction of KPIs has shown improved practice in this area. Monthly pre-hospital blood transfusion KPI analysis now forms part of our rolling governance programme.

## 20) Highest possible ambulatory speed using Lokomat gait training for individuals with a motor-complete spinal cord injury; a clinical pilot study.

### Lysanne van Silfhout^1^, Zdenĕk Váňa^2^, Jakub Pĕtioký^2^, Michael J.R. Edwards^3^, Ronald H.M.A. Bartels^4^, Henk van de Meent^5^, Allard J.F. Hosman^6^

#### ^1^Department of Surgery, Radboudumc, Nijmegen, the Netherlands; ^2^Department of Rehabilitation, Rehabilitation Centre Kladruby, Kladruby, Czech Republic; ^3^Department of Trauma surgery, Radboudumc, Nijmegen, the Netherlands; ^4^Department of Neurosurgey, Radboudumc, Nijmegen, the Netherlands; ^5^Department of Rehabilitation, Radboudumc, Nijmegen, the Netherlands; ^6^Department of Orthopedic Surgery, Radboudumc, Nijmegen, the Netherlands

##### **Correspondence:** Lysanne van Silfhout (Lysanne.vanSilfhout@Radboudumc.nl)

**Background**

Motor impairment and loss of ambulatory function are major consequences of a spinal cord injury (SCI).[1] An exoskeleton is a robotic device that allows SCI patients with limited ambulatory function to ambulate. Mean walking speed of a SCI patient with an exoskeleton is 0.26 m/s.[1] Literature shows that a minimum speed of 0.59 m/s is required to replace wheelchairs in the community.[2] This study aims to investigate the highest ambulatory speed that SCI people can endure, with emphasis on physical factors such as endurance, pain and spasticity. Therefore a Lokomat, which is a gait training device using a treadmill, was used in this study instead of an exoskeleton to eliminate other factors that influence walking speed in an exoskeleton such as balance issues.

**Methods**

This clinical pilot study took place in the Rehabilitation Centre Kladruby, in Kladruby (Czech Republic), a large rehabilitation center which has a Lokomat since 2009. Six motor-complete SCI people were recruited. During a 30 minute Lokomat training, measurements were taken at baseline and directly after finishing the training. Highest achieved walking speed, vital parameters (respiratory frequency, heart rate, blood pressure), Visual Analog Scale (VAS) for pain and Ashworth Scale for spasticity were recorded for each person.

**Results**

The highest reached walking speed in the Lokomat for motor-complete SCI persons was on average 0.63 m/s (SD 0.03 m/s). No negative effects on vital parameters, pain or spasticity were observed. Significant decrease in pain and spasticity after the Lokomat training was observed; 95% CI, 0.336 to 1.664, p=0.012.

**Conclusion**

This study showed that it is possible for motor-complete SCI individuals to ambulate faster than is currently possible with exoskeletons. No negative effects on vital parameters, pain and spasticity was observed while ambulating with on average 0.63 m/s on a Lokomat.

**References**

1. Louie DR, Eng JJ, Lam T. Gait speed using powered robotic exoskeletons after spinal cord injury: A systematic review and correlational study. J Neuroeng Rehabil. 2015;12(1):1–10.

2. Van Silfhout L, Hosman AJF, Bartels RHMA, Edwards MJR, Abel R, Curt A, et al. Ten Meters Walking Speed in Spinal Cord-Injured Patients: Does Speed Predict Who Walks and Who Rolls? Neurorehabil Neural Repair. 2017;31(9):842-850

## 21) Comparison of haemodynamic effects of propofol and esketamine in prehospital intubation

### Risto Viitanen^1^; Jussi Pirneskoski^2^, Ann Cristine Lindroos^3^, Susanne Ångerman^2^, Jouni Nurmi^2^

#### ^1^FinnHEMS Ltd, Research and Development Unit, Vantaa, Finland; ^2^ Emergency Medicine, University of Helsinki and Department of Emergency Medicine and Services, Helsinki University Hospital, Helsinki, Finland; ^3^ Department of Anaesthesia, Helsinki University Hospital, Helsinki, Finland

##### **Correspondence:** Risto Viitanen (risto.viitanen@hus.fi)

**Background**

Prehospital staff is required to intubate critically ill patients in suboptimal conditions and time constraints often prevent haemodynamic optimization before intubation. Currently, haemodynamically most stable anaesthetic for prehospital rapid sequence intubation (RSI) is not known. Ketamine has been suggested as an induction agent instead of propofol due to its more favourable haemodynamic effects. (1,2,3) No studies exist in current literature comparing the haemodynamic effects of esketamine and propofol. Our goal was to compare these two anaesthetics’ influence on hemodynamics during prehospital RSI.

**Methods**

The study was a retrospective observational study using prospectively collected Helsinki University Hospital FinnHEMS 10 (FH10) airway registry data. We included all patients that underwent intubation during years 2014-2016 (N=1071). A protocol for RSI with esketamine as a standard induction agent was implemented in 2015. Before induction was not standardized and propofol was used most commonly. After exclusion (age under 18 years, unknown national social security number, intubated because cardiac arrest, missing vital signs or multiple induction agents used) we divided patients in to two groups according to induction agent used: esketamine (N=373) and propofol (N=179). We used a regression model to evaluate the risk for 30% decrease of systolic blood pressure during induction.

**Results**

There was a greater risk for 30% decrease in systolic blood pressure when using propofol rather than esketamine as an induction agent, OR 1.89 (95% CI 1.30-2.73). The use of fentanyl significantly increases the probability of 30% decrease in systolic blood pressure in both groups, OR 68.81 (95% CI 13.37-379.75). There were no differences between the groups for systolic blood pressure under 90 mmHg post-induction.

**Conclusions**

The use of propofol during prehospital rapid sequence intubation can further compromise haemodynamics in already haemodynamically unstable patients. The use of fentanyl during induction significantly increases the risk of low systolic blood pressure.

**References**

1. Lyon RM, Perkins ZB, Chatterjee D, Lockey DJ, Russell MQ. Significant modification of traditional rapid sequence induction improves safety and effectiveness of pre-hospital trauma anaesthesia. Crit Care. 2015 Apr 1;19(1):617–11.

2. Baekgaard JS, Eskesen TG, Sillesen M, Rasmussen LS, Steinmetz J. Ketamine as a Rapid Sequence Induction Agent in the Trauma Population: A Systematic Review. Anesthesia & Analgesia. 2018 Jun 25;:1–7.

3. Sahinovic MM, Struys MMRF, Absalom AR. Clinical Pharmacokinetics and Pharmacodynamics of Propofol. Clinical Pharmacokinetics. Springer International Publishing; 2018 Jul 17;26(Suppl 4):1–20.

## 22) Could intubate, but can’t ventilate through a blocked tube. Now what?

### Espen Fevang

#### Department of Research and Development, Norwegian Air Ambulance Foundation, Oslo, Norway

##### **Correspondence:** Espen Fevang (espen.fevang@norskluftambulanse.no)

**Background**

Vomit and blood obstructing the airway has been reported as the most common complication when trained personnel perform endotracheal intubation (ETI) in a pre-hospital setting [1]. Aspiration of gastric contents prior to ETI may cause food solids to block the tube after correct placement, preventing ventilation. A technique to overcome this obstacle was successfully used in a real-life setting.

**Case report**

After a submersion accident involving two children in cardiac arrest, ETI during regurgitation was achieved with the patients in a semiprone position. This allowed for a constant drainage of gastric and pulmonary contents and was later shown in a simulation study to be a feasible technique under such circumstances [2]. After successful intubations, both tubes became blocked by remaining food solids too large to be removed by suction through the tubes, obstructing expiration and preventing regular ventilations. This created a potentially detrimental situation, as the children were in an immediate need of oxygenation due to hypoxic cardiac arrest. By deflating both patients tube cuffs and giving short and rapid ventilations during chest compressions, expiration and food solids could pass outside the tubes while maintaining oxygenation and ventilation. This improvised jet-ventilation resolved the situation and after approximately ten minutes the lungs had cleared sufficiently to allow normal bag ventilation. Resuscitation was successful and neither of the children experienced pneumonia or acute respiratory distress syndrome during their stay in the intensive care unit. Both children were discharged from hospital with no known sequelae.

**Conclusion**

By deflating the endotracheal tube cuff and giving rapid, short ventilations during chest compressions, objects too large to pass through the endotracheal tube may pass on the outside, allowing for ventilation and oxygenation with a simultaneous clearing of a soiled airway.

**References**

1. Helm M, Hossfeld B, Schafer S, Hoitz J, Lampl L. Factors influencing emergency intubation in the pre-hospital setting--a multicentre study in the German Helicopter Emergency Medical Service. Br J Anaesth. 2006;96(1):67-71.

2. Fevang E, Haaland K, Roislien J, Bjorshol CA. Semiprone position is superior to supine position for paediatric endotracheal intubation during massive regurgitation, a randomized crossover simulation trial. BMC Anesthesiol. 2018;18(1):10.

Written informed consent was obtained from the patients’ parents for publications addressing patient information in the described incident.

## 23) 6 months of Code Red Trauma – can we improve the management of traumatic haemorrhage?

### Pip Lewis^1^, Nicola Batick^1^, Denise Mckeown^2^, Fateha Chowdhury^2^, Chris Aylwin^3^

#### ^1^Emergency Department, St Mary’s Hospital, Imperial College NHS Healthcare Trust, London, UK; ^2^Haematology Department, St Mary’s Hospital, Imperial College NHS Healthcare Trust, London, UK; ^3^Vascular Surgery Department, St Mary’s Hospital, Imperial College NHS Healthcare Trust, London, UK

##### **Correspondence:** Pip Lewis (pip.lewis1@nhs.net)

**Background**

Major haemorrhage remains a potentially treatable cause of death from major trauma. This study aims to explore the prevalence, patient characteristics, patterns of blood product use and outcomes from major haemorrhage in trauma at an urban Major Trauma Centre.

**Methods**

A review of 6 months of ‘Code Red’ major trauma calls from Jan 2018 – June 2018 was carried out. Data was obtained by reviewing trauma notes, transfusion charts and blood bank records.

**Results**

In the 6-month period there were 65 declared ‘Code Red’ cases, of which 55 received blood products. Mean ISS was 33 (9-75) with 89.1% of cases having an ISS >15. 50.9% of cases were secondary to blunt trauma. The overall mortality was 18.2%. Mortality from blunt trauma was higher than that of penetrating (20.7% versus 11.5%. 25.5% of cases underwent massive transfusion (>10 units RBC in 24 hours) of which 35.8% died. Patients received a mean of 8.4 units of packed red cells, 6.1 units of FFP and 1 unit of platelets in the first 24 hours of admission. The mean ratio of FFP to PRC was 0.85. Mean ionised calcium on initial venous blood gas was 1.05mmol/L and mean fibrinogen level 2.48g/L. Initial tranexamic bolus was given to all patients, but the second infusion was only documented as given in ED in 41.8% of cases.

**Discussion**

The overall mortality from Code Red trauma in this study group is comparable to previously published research [1,2], and has improved when compared to previous audits from this MTC. Improved outcomes may be related to the availability of pre-thawed FFP and pre-hospital transfusion. There is work to be done to improve compliance and recording of compliance with 1:1:1 protocols as well as the use of calcium, tranexamic acid and thromboelastography, particularly in the Emergency Department.

**References**

^1^Stanworth SJ, Davenport R. Mortality from trauma haemorrhage and opportunities for improvement in transfusion practice. Br J Surg. 2016 Mar;103(4):357-65

^**2**^Sinha R^1^, Roxby D**.** Experience with a massive transfusion protocol in the management of massive haemorrhage. Transfus Med. 2013 Apr;23(2):108-13.

## 24) The patterns of point of care ultrasound use by an Australian pre-hospital retrieval medicine service

### Philip Bewley^1^, Matthew Miller^2^, Christopher Partyka^2^

#### ^1^University Hospitals Bristol, Bristol, UK; ^2^Greater Sydney Area HEMS, Sydney, Australia

##### **Correspondance**: Philip Bewley (bewleyphilip@gmail.com)

**Background**

Pre-hospital ultrasound is widely used. [1] Evidence supports its feasibility and usefulness, particularly in trauma. [2] Despite this, there has been no study examining the patterns of point of care ultrasound use (POCUS) in an Australian retrieval service for ten years. [3] GSA-HEMS is a pre-hospital and retrieval medicine service serving critically ill and injured patients in New South Wales, Australia. We report on the patterns of POCUS use in this service over a five-year period.

**Method**

A retrospective database review was performed. Patients who were attended to by a GSA-HEMS team and received a POCUS study between January 1^st^ 2013 and December 31^st^ 2017 were included.

**Results**

12930 missions were reviewed. POCUS was used in 2954 missions (23%). POCUS is more commonly used during pre-hospital missions. Use increased from 30% in 2013 to 51% in 2017.eFAST is the most commonly performed scan. 53% were complete. Pleural fluid, pelvic and LUQ components are most likely to be incomplete or indeterminate. Perceived impact of POCUS on scene time was self-reported. 89% reported no effect or a reduction of POCUS on scene time.

**Discussion**

Increased use of ultrasound likely represents improvements in technology and accessibility of training. The percentage of incomplete scans is concerning. Information that alters patient management may be missed. Current database design prevents analysis of why patients are not scanned. We plan to alter database documentation to better record this information. We also plan to provide additional teaching focusing on eFAST problem areas and overcoming specific pre-hospital difficulties e.g. scanning the LUQ in a helicopter.

This was a large retrospective study examining an area of practice that has not been assessed for ten years. The increased use of PHUS is encouraging. We have identified areas of practice that can be improved that are relevant to HEMS services.

**References**

**1.** Nelson BP, Sanghvi A. Out of hospital point of care ultrasound: current use models and future directions. European Journal of Trauma and Emergency Surgery. 2016; 42: 139-50

2. O'Dochartaigh D, Douma M. Prehospital ultrasound of the abdomen and thorax changes trauma patient management: A systematic review. Injury. 2015; 46: 2093-102.

3. Mazur SM, Pearce A, Alfred S, Sharley P. Use of point-of-care ultrasound by a critical care retrieval team. Emergency Medicine Australasia. 2007; 19: 547-52.

## 25) ECMO in major trauma: a systematic literature review

### Samuel Nava^1^, Marius Rehn^2^, Julian Thompson^1^

#### ^1^ North Bristol NHS Trust, Bristol, UK; ^2^ Norwegian Air Ambulance Foundation, Oslo, Norway

##### **Correspondence:** Samuel Nava (samuelnava@doctors.org.uk)

**Background**

ECMO (extra-corporeal membrane oxygenation) performed in the major trauma population presents unique challenges and opportunities [1]. We undertook a systematic literature review to assess the breadth of roles for ECMO in major trauma and evaluate the evidence base behind its use.

**Methods**

We carried out a comprehensive search of the following databases: MEDLINE, PubMed, EMBASE, CENTRAL and CINAHL. The search included adult patients in receipt of all forms of ECMO support in any form at any stage during their care for injuries sustained as a direct result of major trauma or for secondary complications

**Results**

The initial search identified 4625 records and following removal of duplicate reports, abstract and full text review, we identified 96 reports exclusively on trauma patients. We found published literature from 1972 to 2018 from 18 different countries. The vast majority of studies were case series or reports with no randomised control trials or case-control studies. Indications for ECMO included respiratory failure, cardiac failure, bleeding shock and peri-operative ECMO. There were a range of injuries and disease pathologies causing organ system failure. ECMO was used in veno-venous and veno-arterial forms usually depending upon the cardiovascular status of the patient. A number of studies report heparin-free ECMO in patients with bleeding shock or traumatic brain injury who may conventionally have been excluded from ECMO therapy. Pooled mortality to hospital discharge in 22 studies of 6 or more patients was 42.2%.

**Discussion**

This study provides a completed picture of published international literature on the use of ECMO in major trauma with a broad range of indications, pathologies and injury patterns. Centres are using VV and VA ECMO tailored to each patient and ECMO is used in the immediate, early and late phases of trauma care.

**References**

1. Zonies D, Merkel M. Advanced extracorporeal therapy in trauma. Curr Opin Crit Care. 2016 Dec;22(6):578-583.

## 26) Search and rescue compared to remote medical evacuation in a Norwegian setting

### Bjørn O Reid ^1^, Helge Haugland ^2^,Marius Rehn ^3^, Oddvar Uleberg ^2^, Andreas J Krüger ^2^

#### ^1^ Norwegian Armed Forces Medical Services, Sessvollmoen, Norway; ^2^ Department of Emergency Medicine and Prehospital Services, St. Olavs hospital, Trondheim, Norway; ^3^ Department of Research and Development, Norwegian Air Ambulance Foundation, Oslo, Norway

##### **Correspondence:** Bjørn O Reid (bjorn.ole.reid@stolav.no)

**Background**

Helicopter emergency medical services (HEMS) may perform Search and Rescue (SAR) missions whereas SAR services also perform medical evacuation (medevac) [1, 2]. This depends on helicopter type, training, crew composition, equipment and local interdisciplinary procedures for cooperation. We aim to describe characteristics of SAR- and remote medevac missions in a military SAR helicopter system compared to a civilian HEMS operating in the same region.

**Materials and methods**

Retrospective, observational study of SAR- and remote medevac missions performed at a Norwegian military SAR helicopter-(Ørland) and civillian HEMS base (Trondheim) during the five-year period from January 1^st^ 2013 to December 31^st^ 2017. Remote medevac terrain was defined as rugged, isolated or water (inland, coastal or offshore). We applied descriptive statistics and Student`s t-test for comparisons.

**Results**

We included 721 missions. The SAR service performed 359 (50%) missions. These constituted 237 (33%) SAR- and 122 (17%) remote medevacs. The HEMS service performed 362 (50%) missions, of which 85 (12%) were SAR- and 277 (38%) were remote medevacs. The mean mission time for SAR was 152 minutes (IQR 100-235) for the SAR service and 57 minutes (IQR 34-89) for HEMS. The SAR service performed 121 (17%) hoist operations compared to 20 (3%) human cargo sling operations by HEMS. The dominating mechanism was trauma in 299 (48%), medical conditions in 134 (21%) and psychiatry in 53 (9%) of included patients (n=624). The most frequently performed procedures were fracture reductions (6%) followed by endotracheal intubation (3%). Remote medevac patients in both services had a higher mean National Advisory Committee for Aeronautics score of 3.16 (95% CI 3.03-3.28) compared to 1.95 (95% CI 1.61-2.28) in SAR missions (*p< 0.05*).

**Conclusion**

Norwegian SAR- and HEMS services perform SAR- and remote medevac missions extensively. Mission profiles between the two services vary.

**References**

1. Kruger AJ, Skogvoll E, Castren M, Kurola J, Lossius HM. Scandinavian pre-hospital physician-manned Emergency Medical Services--same concept across borders? Resuscitation. 2010;81(4):427-33.

2. Glomseth R, Gulbrandsen FI, Fredriksen K. Ambulance helicopter contribution to search and rescue in North Norway. Scandinavian journal of trauma, resuscitation and emergency medicine. 2016;24(1):109.

## 27) Prehospital blood transfusion: a comparison of non-trauma and trauma patients

### Susanne Ångerman^1^, Katja Salmela^2^, Hetti Kirves^3^, Jouni Nurmi^1^

#### 1 Emergency Medicine and Services, Helsinki University Hospital and University of Helsinki, Helsinki, Finland; 2 Hospital Blood Bank, Helsinki University Hospital laboratory (HUSLAB), Helsinki University Hospital (HUS), Helsinki, Finland; 3 Prehospital Emergency Care, Hyvinkää hospital area, Hospital District of Helsinki and Uusimaa, Hyvinkää, Finland

##### **Correspondence:** Susanne Ångerman ( susanne.angerman-haasmaa@hus.fi)

**Background**

Prehospital blood transfusion (PHBT) for trauma patients with major bleeding is an established intervention in many services [1,2]. Literature on the use of blood products for non-traumatic patients in prehospital setting is limited [3]. We aimed to compare non-trauma and trauma patients receiving PHBT with similar hemodynamic triggers.

**Method**

PHBT protocol including two packed red blood cells (pRBC) and freeze-dried plasma (FDP) units was implemented in one medical helicopter unit in 2016. The triggers for transfusion were strong clinical suspicion of massive hemorrhage and systolic blood pressure below 90 mmHg or absent radial pulse. We performed a retrospective review of prospectively collected quality registry data of two years.

**Results**

Total of 27 non-trauma patients and 57 trauma patients received PHBT. Non-trauma patients were elder (62±18 vs. 43±20, P<0.001) and 19 (70%) of these were male compared to 47 (82%) of trauma patients (NS). Non-trauma group included patients with gastrointestinal bleeding (10), vascular catastrophe (8), post-operative bleeding (4), gynecological/obstetrical bleeding (2) and other nontraumatic hemorrhages (3). No difference was detected in the number of administered pRBCs, FDP units or the volume of given crystalloids between the groups. Cardiac arrest occurred in 6 non-trauma and 11 trauma patients during prehospital care, of which 3 and 8 patients survived to hospital with spontaneous circulation. On hospital admission the non-trauma patients were characterized with lower hemoglobin (97±22 vs. 120±19 g/l, P<0.0001), higher pH (7.32±0.18 vs. 7.24±0.17, P=0.02) and lower plasma thromboplastin time (55±24 vs. 71±23%, P=0.02). Blood products were not needed after hospital admission in 4/24 (17%) and 19/54 (35%) in non-trauma and trauma patients, respectively.

**Conclusion**

We described a heterogenic patient group with massive hemorrhage. Non-trauma patients in need of prehospital blood products seem to be slightly older and at least as critical as trauma patients.

**References**

1. Shackelford SA, Del Junco DJ, Powell-Dunford N, et al. Association of Prehospital Blood Product Transfusion During Medical Evacuation of Combat Casualties in Afganistan With Acute and 30-Day Survival. JAMA. 2017; 318:1581-1591

2. Sperry JL, Guyette FX, Brown JB, et al. Prehospital Plasma during Air Medical Transport in Trauma Patients at Risk for Hemorrhagic Shock. N Engl J Med. 2018; 379:315-326

3. Thiels CA, Aho JM, Fahy AS, et al. Prehospital Blood Transfusions in Non-Trauma Patients. World J Surg. 2016; 40:2297 – 2304

## 28) Neurosurgical clinicians’ awareness of current Driver and Vehicle Licensing Agency (DVLA) standards

### Paulette Y O Kumi, Alice Kershberg, Davor Dasic, Christopher E G Uff

#### Neurosurgery, Royal London Hospital, London, UK

##### **Correspondence:** Paulette Y O Kumi (paulette.kumi@nhs.net)

**Background**

18% of world’s burden of disease is due to injury (1). Despite efforts to control this – including efforts such as the 2030 Agenda for Sustainable Development is a target of halving the global number of deaths and injuries from road traffic crashes by 2020 -, Injury related deaths expected to rise dramatically by 2020 and it is projected to rise by 80% in low and middle-income countries (1). A concerted effort on our part as clinicians is thus required. As the eye cannot see what the mind does not know, we decided to assess our awareness of clinicians of current DVLA guidance (2, 3) and provide a forum to review the current guidance (4, 5) and discuss ways to better engage with this guidance.

**Methodology**

Paper based questionnaire with the following questions

1. Neurosurgical requiring DVLA notification or requiring driving restrictions

2. When should DVLA be notified about intracranial tumor

3. Can DVLA be notified without patient consent where persistent unintentional compliance is a concern.

The participants were neurosurgical clinical staff (33 doctors and senior nurses)

**Results**

37% were up to date with current regulations

20% were unsure of if disclosure could be made to DVLA in the context of intentional noncompliance

10% answered no to if clinicians can disclose in cases of persistent intentional non-compliance.

**Conclusion**

There is a significant lag between changes to DVLA requirements and implementation in clinical practice. There is a need to explore more ways for the DMG to engage clinicians in the field.

**References**

1. https://www.who.int/violence_injury_prevention/road_safety_status/2018/en/

2. https://www.gov.uk/.../assessing-fitness-to-drive-a-guide-for-medical-professionals

3. http://pediatrics.aappublications.org/content/132/1/4

4. Thomas S, Mehta M, Kuo JS, Robins I, Khuntia D (2010) Current practices of driving restriction implementation for patients with brain tumors. J Neurooncol. doi:10.1007/s11060-010-0439-7 [PubMed]

5. Chin YS, Jayamohan J, Clouston P, Gebski V, Cakir B. Driving and patients with brain tumours: a postal survey of neurosurgeons, neurologists and radiation oncologists. J Clin Neurosci. 2004;11:471–474. doi: 10.1016/j.jocn.2003.08.010.

## 29) Validation of the Dutch clinical prediction rule for ambulation outcomes in an inpatient setting following traumatic spinal cord injury

### Lysanne van Silfhout^1,2^, Annemieke EJ Peters^1,2^, Marnie Graco^1^, Rachel Schembri^1^, Andrew K Nunn^2^, David J Berlowitz^1^

#### ^1^Institute for Breathing and Sleep, Austin Health, Heidelberg, Melbourne, Victoria, Australia; ^2^Victorian Spinal Cord Service, Austin Health, Heidelberg, Melbourne, Victoria, Australia

##### **Correspondence:** Lysanne van Silfhout (Lysanne.vanSilfhout@Radboudumc.nl)

**Background**

A clinical prediction rule for ambulation outcomes after a traumatic spinal cord injury (SCI) has been published by van Middendorp et al.[1] This Dutch prediction rule is reported to provide a highly accurate and early prognosis of a patient’s ability to walk at 1 year post injury, using age, motor scores of the quadriceps femoris (L3), gastrocsoleus (S1) muscles, and light touch sensation of dermatomes L3 and S1. However, the accuracy of this prediction rule has only been established within the original Dutch study that involved specifically trained neurologists and rehabilitation physicians. Therefore the aim of this study is to determine the accuracy of a previously described Dutch clinical prediction rule for ambulation outcome in routine clinical practice.

**Methods**

We included adults (≥ 18 years) who were admitted to the Austin Hospital with a traumatic SCI between January 2006 and August 2014. Data from medical records were collected retrospectively to determine the score of the Dutch clinical ambulation prediction rule as described by van Middendorp et al.[1] A receiver-operating characteristics (ROC) curve was generated to investigate the performance of the prediction rule. Univariate analyses were performed to investigate which factors significantly influence ambulation after a traumatic SCI.

**Results**

The area under the ROC curve (AUC) obtained during the current study (0.939, 95% confidence interval (CI) (0.892, 0.986)) was not significantly different from the AUC from the original Dutch study (0.956, 95% CI (0.936, 0.976)). Factors that were found to have a significant influence on ambulation outcome were time spent in the ICU, number of days hospitalised and injury severity.

**Conclusion**

The Dutch ambulation prediction rule performed similarly in routine clinical practice as in the original, controlled study environment in which it was developed.

**Reference**

1. van Middendorp JJ, Hosman AJ, Donders AR, Pouw MH, Ditunno JF Jr, Curt A et al. A clinical prediction rule for ambulation outcomes after traumatic spinal cord injury: a longitudinal cohort study. Lancet 2011; 377: 1004–1010.

## 30) The influence of time on the predictive value of the post-resuscitation electrocardiogram: a single centre, retrospective, observational pilot study

### Carl Evans^1^, Dr Magnus Nelson^2^

#### ^1^Brighton and Sussex Medical School; ^2^Brighton and Sussex University Hospitals

##### **Correspondence:** Carl Evans (carl.t.evans@me.com)

**Background**

Following pre-hospital return of spontaneous circulation (ROSC), the electrocardiogram (ECG) is used to decide whether a patient has suffered a coronary occlusion which would benefit from immediate primary coronary intervention (PCI) or whether the patient should go to the Emergency Department (ED). Numerous studies have cast doubt over the reliability of the post-ROSC ECG as defibrillation [1], myocardial hypoperfusion and reperfusion injury [2] have all been shown to cause misleading results. However, this artefact may resolve over time as myocardial perfusion is restored.

**Methods and Results**

A 1-year single-centre, retrospective, observational analysis sought post-ROSC patients who underwent coronary angiography and had a pre-hospital and delayed-hospital post-ROSC ECG available for analysis. 42 Post-ROSC ECGs were interpreted and positive and negative results were viewed alongside angiographic findings to calculate the predictive values of the post-ROSC ECGs.

The pre-hospital ECG had a sensitivity of 25%, specificity of 60%, positive predictive value of 66% and a negative predictive value of 20% for predicting a clinically significant coronary occlusion, with an overall accuracy of 33%. In comparison, the delayed-hospital post-ROSC ECG had a sensitivity of 69%, specificity of 100%, positive predictive value of 100% and a negative predictive value of 50%, with an overall accuracy of 76%.

Classifying the post-ROSC ECG predictions as either ‘correct’ or ‘incorrect’ demonstrated that the delayed post-ROSC ECG was statistically significantly more accurate in predicting a causative coronary occlusion, (chi-squared value 7.78, p=0.0053, p<0.05).

**Conclusions**

These results suggest that it may not be possible to triage the pre-hospital post-ROSC patient to either ED or PCI, on the basis that at this initial stage it is truly unknown what definitive care is required. Instead, a compromise may be required where a patient is conveyed to an ED with co-located PCI facilities, reinforcing the need for regional ‘cardiac arrest centres’.

**References**

1. Shan P, Lin J, Xu W, Huang W. ST-segment elevation after direct current shock mimicking acute myocardial infarction: a case report and review of the literature. The American journal of emergency medicine [online]. 2014 Nov 1 [accessed 2018 Nov 2];32(11):1438-e1. Available from: https://www.ajemjournal.com/article/S0735-6757(14)00245-9/abstract

2. Stær-Jensen H, Nakstad ER, Fossum E, Mangschau A, Eritsland J, Drægni T, Jacobsen D, Sunde K, Andersen GØ. Post-resuscitation ECG for selection of patients for immediate coronary angiography in out-of-hospital cardiac arrest. Circulation: Cardiovascular Interventions [online]. 2015 Oct 1 [accessed 2018 Nov 2];8(10):e002784. Available from: https://www.ahajournals.org/doi/abs/10.1161/circinterventions.115.002784

## 31) The predictive value of the post-resuscitation electrocardiogram: a review

### Carl Evans^1^, Dr Gareth Grier^1,2^, Professor Tim Harris^3^

#### ^1^The Institute of Pre-Hospital Care, Whitechapel, London, UK; ^2^London’s Air Ambulance, Whitechapel, London, UK; ^3^The Royal London Hospital, Whitechapel, London, UK

##### **Correspondence:** Carl Evans (carl.t.evans@me.com)

**Background & Aim**

Recent studies have described post-resuscitation patients who have been found to have suffered an acute myocardial infarction (AMI) despite no ST-elevation on their post-resuscitation electrocardiogram [1]. Many authors suggest a lack of ST-elevation on the post-resuscitation electrocardiogram cannot be used to exclude an AMI and every patient should receive percutaneous coronary intervention, regardless of their post-resuscitation electrocardiogram [2]. However, this isn’t possible in many areas due to limited resources.

The aim of this review was to collate and appraise the evidence describing the predictive values of common post-resuscitation electrocardiogram patterns to indicate which patients would benefit from percutaneous coronary revascularisation.

**Methods and Results**

A search of PubMed, Embase and Web of Science was conducted for all years with an aim of identifying studies describing post-resuscitation electrocardiogram patterns and associated angiographic findings in adult patients who had been resuscitated from a cardiac arrest. Predictive values of common post-resuscitation electrocardiogram changes were collated and reviewed to identify any patterns predictive of the need for percutaneous coronary revascularisation. 9 observational studies were included for analysis. ST-elevation, ST-depression and left bundle branch block were the most common post-resuscitation electrocardiogram patterns for patients suffering an AMI.

**Discussion and Conclusion**

Post-resuscitation patients rarely have normal electrocardiograms, regardless of underlying coronary pathology. Whilst ST-elevation is commonly associated with underlying coronary occlusions, this review has reinforced the suggestion that the absence of ST-elevation cannot be used to rule out the need for coronary intervention, as other electrocardiogram patterns can be predictive of coronary occlusions. However, instead of performing coronary intervention on all post-resuscitation patients, the combination of ST-elevation and/or ST-depression and/or left bundle branch block as a triage tool is able to detect the majority of patients with an underlying AMI (even in the absence of ST-elevation) with a 98% sensitivity and a 53% specificity.

**References**

1. Zanuttini D, Armellini I, Nucifora G, Grillo MT, Morocutti G, Carchietti E, et al. Predictive value of electrocardiogram in diagnosing acute coronary artery lesions among patients with out-of-hospital-cardiac-arrest. Resuscitation. 2013 Sep;84(9):1250–4.

2. Dumas F, Cariou A, Manzo-Silberman S, Grimaldi D, Vivien B, Rosencher J, et al. Immediate percutaneous coronary intervention is associated with better survival after out-of-hospital cardiac arrest: Insights from the PROCAT (Parisian Region Out of Hospital Cardiac Arrest) registry. Circ Cardiovasc Interv. 2010;3(3):200–7.

## 32) Introducing Cardiopulmonary Resuscitation Teaching in a Secondary School in the UK

### Isobel Abbott^1^, Julia Neely^2^, Alick Robertson^1^

#### ^1^ Sweyne Park School, Rayleigh, Essex, UK; ^2^ Department of Anaesthetics, Broomfield Hospital, Chelmsford, Essex, UK

##### **Correspondence:** Julia Neely (julia.neely@cantab.net)

**Background**

Initiatives to promote children learning cardiopulmonary resuscitation (CPR) have been introduced internationally^[1]^. Mandating CPR teaching in elementary schools, as part of a multi-faceted approach to improve Danish out-of-hospital cardiac arrest outcomes, has seen survival triple over a decade^[2]^. The United Kingdom will be including CPR teaching in national school curricula by 2020. We wanted to assess the feasibility of delivering Basic Life Support (BLS) training to a Year 7 cohort (aged 11 to 12 years), and to assess baseline CPR knowledge in Sixth Form students (16 to 18 years). This latter group will not benefit from future government education plans.

**Method**

The Year 7 cohort was divided into three one-hour sessions of approximately 90 students. Following a lecture and demonstration, each child simulated BLS as part of a group using 15 low-fidelity resuscitation mannequins. They were supervised and given feedback throughout this process, before proceeding to a reflective discussion regarding bystander CPR. The Sixth Form students were provided with a survey to complete prior to undertaking their session.

**Results**

268 students in Year 7 were trained in CPR over the course of a day. Sixth Form survey results demonstrated 38% of students had undertaken prior CPR training. However, all students were not confident in resuscitating an adult or child. Only 1 student could identify all scenarios requiring CPR from a range of options. All considered the session would be fairly or very relevant to them.

**Discussion**

It is possible to provide BLS training to a large cohort of secondary school students, without significant resource requirements. We have highlighted a lack of understanding and knowledge of CPR within our Sixth Form students, consistent with findings elsewhere^[3]^. A repeat survey will be conducted to determine whether information from the initial CPR session has been retained.

Written, informed consent was obtained from all Sixth Form students.

**References**

1 Kids Save Lives [https://www.kids-save-lives.eu/], accessed online 3^rd^ November 2018.

2 Wissenberg M, Lippert F, Folke F. Association of national Initiatives to Improve Cardiac Arrest Management with Rates of Bystander Intervention and Patient Survival After Out-of-Hospital Cardiac Arrest. JAMA. 2013;310(13):1377-1384.

3 Aaberg A, Larsen C, Rasmussen B, Hansen C, Larsen J. Basic life support knowledge, self-reported skills and fears in Danish high school students and effect of a single 45-min training session run by junior doctors; a prospective cohort study. Scand J Trauma Resusc Emerg Med. 2014;22:24-29.

## 33) A novel graphical method for displaying anatomical injury patterns caused by different mechanisms of injury in 11,285 pedal cyclists

### Rowena Johnson^1^, Nick Dodds^1^, Benjamin Walton^1^, Bobby Stuijfzand^2^, Julian Thompson^1^

#### ^1^ Critical Care, North Bristol NHS Trust, Bristol, UK; ^2^ University of Bristol Jean Golding Institute, Bristol, UK

##### **Correspondence:** Rowena Johnson (rowena.johnson@nbt.nhs.uk)

**Background**

Despite recent improvements in road architecture and safety, 18,321 injuries and 6% of road deaths in the UK during 2017 were pedal cyclists[1]. Early injury prediction from mechanism of injury has been proposed as improving patient treatment and triage[2,3]. This study evaluated a national cycle injury dataset to determine whether specific collision types could be graphically presented to assist injury pattern prediction and guide management.

**Method**

The Trauma Audit and Research Network (TARN) database was retrospectively interrogated to identify all adult (≥16 years) patients presenting to hospital with cycling-related injuries, during a period from 14 March 2012 to 30 September 2017. We identified patient demographics, mechanism of injury, cycle protection (helmet use) and outcomes. Mechanism of injury was grouped as: collision with stationary object, vs large vehicle, vs small vehicle, non-collision fall from bicycle, off road, and unknown. R, a statistical package, was used to analyse and display data so that pattern of injury could quickly be predicted from patient demographics and mechanism of trauma.

**Results**

11285 cyclists were included in the study. Injury pattern varied with age, with higher rates of chest wall injury in older cyclists, and increased incidence of head injury in younger cyclists. Abdominal and spinal trauma was uncommon, but more likely to occur when off road cycling. The greatest burden of injury occurred following large vehicle collision, which frequently resulted in severe pelvic and leg injuries and was associated with the highest mortality of all mechanisms of injury.

**Discussion**

We have displayed the data graphically in a way that could allow for rapid prediction of injury pattern and guide management. Further research needs to be conducted to construct a real time interface to assist clinicians in giving appropriate care based on available data at the time of injury.

**References**

1. Department for Transport. Reported road casualties in Great Britain: 2017 annual report.

2. Wilmer I, Chalk G, Davies GE, et al Air ambulance tasking: mechanism of injury, telephone interrogation or ambulance crew assessment? Emerg Med J 2015;32:813-816.

3. Neumann MV, Eley R, Vallmuur K, Schuetz M. Current profile of cycling injuries: A retrospective analysis of a trauma centre level 1 in Queensland. Emerg Med Australas. 2016;28:90-95

## 34) Case feedback requests from pre-hospital practitioners - what do they want to know?

### Andrew Patton, David Menzies

#### Emergency Department, St. Vincent’s University Hospital, Dublin, Ireland

##### **Correspondence:** Andrew Patton (andrew@pattons.ie)

**Background**

It can be challenging for Pre-Hospital Practitioners to follow-up on patients they treat and transport to hospital.[1] Clinical follow-up and feedback are necessary to improve diagnostic and clinical skills.[2] In August 2017 we set-up a pre-hospital feedback service at a university hospital Emergency Department (ED) using a ‘*Pre-Hospital Post-Box’*. Our ED had 56,541 patient presentations during the study period, 17,469 of which presented by ambulance.

**Methods**

We reviewed all the feedback request forms received over a 12 month period from 1st September 2017 - 31st August 2018. The data was extracted from the forms, recorded on a spreadsheet and analysed in Microsoft Excel 2011.

**Results**

282 feedback requests (1.6% of total ambulance presentations) were received from 116 pre-hospital practitioners (average 2.4 requests per person, range 1-16). Student or Intern Paramedics initiated 49% of requests, Advanced Paramedics (28%) and Paramedics (22%).

Trauma (19%), collapse (15%), respiratory difficulties (12%), chest pain (12%) and neurological symptoms (7%) were most the most common presentations where feedback was requested.

57% of requests related to male patients, 12% of patients were aged <35 years, 44% (35-65 years) and 44% (>65 years).

The vast majority of requests were regarding the diagnosis, outcome and ED management of the patient, 4% enquired about ECG or blood results, and 9% radiology results. Only 7% of requests asked a specific question related to the case.

**Conclusion**

Pre-hospital practitioners are interested to learn the diagnosis and outcome of patients they treat, in particular cases where the diagnosis can’t be confirmed in the pre-hospital setting or where further investigations are required. The cases where feedback was sought are representative of the total ambulance presentations to our ED in terms of demographics and pathology, suggesting Paramedic factors rather than the patient or illness may be the predominant reason for seeking feedback.

**References**

1) Jenkinson E, Hayman T, Bleetman A. Clinical feedback to ambulance crews: supporting professional development. Emerg Med J. 2009;26:309.

2) Croskerry P. The feedback sanction. Acad Emerg Med. 2000;7:1232-8.

## 35) Feedback for pre-hospital practitioners – a quality improvement initiative

### Andrew Patton, David Menzies

#### Emergency Department, St. Vincent’s University Hospital, Dublin, Ireland

##### **Correspondence:** Andrew Patton (andrew@pattons.ie)

**Background**

Feedback is important for clinicians to improve their diagnostic performance through calibration.[1] Prior to the introduction of our pre-hospital feedback service, there were no formal structures in Ireland to allow pre-hospital practitioners receive feedback on patients they treat. To address this, we commenced a service development project.

**Methods**

A standard operating procedure was developed to govern the feedback process. This addressed issues including confidentiality, consent and data protection. Subsequently we consulted *Ysbyty Gwynedd* Emergency Department in Bangor, Wales, where a similar system was already in place. Using templates from Bangor, we created a feedback request form. These were made available in the ‘Ambulance Triage’ area of our Emergency Department (ED), and posters advertising the new service were displayed. Requests submitted were reviewed by a designated Emergency Medicine (EM) Doctor and feedback was provided to the practitioner by phone.

**Results**

282 feedback requests were received in the first 12 months (September 2017 - September 2018). The average time from patient presentation to feedback being provided was 23 days (Range 0 - 108 days). 37% of feedback requests were answered within 2 weeks, 66% within 1 month and 83% within 6 weeks. 10 minutes was the average time required to collate details for the feedback request (reviewing the ED chart, radiology, labs and discharge letters), and 4-5 minutes per phone call. A follow-up survey indicated a high level of satisfaction with the feedback process among pre-hospital staff. 83% reported the feedback to be very comprehensive and 100% reported that patient follow-up benefits their clinical knowledge and practice

**Conclusion**

We have developed a formal process to provide confidential feedback to pre-hospital practitioners regarding patients they treat. It is labour intensive, but we believe it is a worthwhile initiative as it enables practitioners to enhance their diagnostic performance and develop their clinical practice.

**Reference**

1) Croskerry P. The feedback sanction. Acad Emerg Med. 2000;7:1232-8.

## 36) Experiences of doctors and London Ambulance Service staff working as part of the Physician Response Unit: a survey of job satisfaction, training opportunities and burnout

### Lisa Ramage, Charlotte Ashworth, Jess Payne, Tony Joy

#### Physician Response Unit, London’s Air Ambulance, Bart’s Health NHS Trust. Royal London Hospital, Stepney Way London E1 1BB

##### **Correspondence:** Tony Joy (Tony.Joy@bartshealth.nhs.uk)

**Background**

The Physician Response Unit (PRU) employs a number of emergency medicine (EM) doctors and London Ambulance Service (LAS) staff through one-year fellowships or secondments. Remodelled in 2017 in response to the Five Year Forward View[1], the PRU responds to undifferentiated 999 calls across Northeast London, and aims to bring the emergency department to the patient. This study aimed to ascertain the experience of PRU doctors and LAS staff in terms of job satisfaction, training opportunities and burnout rates.

**Method**

An anonymised questionnaire was designed using open-ended questions and 5-point Likert Scales. Burnout data was collated using a modified version of the Maslach Burnout Inventory Scale[2], scored in three domains: emotional exhaustion (EE; maximum score 45), depersonalisation (DP; maximum score 25) and personal achievement (PA; maximum score 40; reverse scored) Mean responses were compared to predefined cut-offs for low/moderate/high scores.

**Results**

15 responses were received (8 doctors, 7 LAS staff). 100% indicated their time with the PRU had changed clinical practice; 85% stated it would influence subsequent career progression. 100% felt inspired by the ethos of the PRU. 93% believed care provided was superior to standard care. 87% reported greater confidence in managing critically unwell patients, cardiac arrests, major trauma, airway emergencies and complex social problems. Most implied greater understanding of challenges faced by ambulance services (100% of doctors) and emergency departments (93%). Mean burnout scores in each domain were indicative of low burnout levels: EE: 11.07+/-7.08; DP: 3.08+/-3.60; PA: 34.80+/-5.95.

**Discussion**

The results demonstrate working on the PRU is influential in future career choices and subsequent clinical practice. Numerous training benefits are provided; these are advantageous for staff and transferrable to their regular working arenas. Provision of split-working schedules such as those provided with these attractive PRU secondments might aid recruitment and retention of frontline emergency staff.

**References**

1. National Health Service. Five Year Forward View. 2014. Available from ttps://www.england.nhs.uk/wp-content/uploads/2014/10/5yfv-web.pdf

2. Chigerwe M, Boudreaux KA, Ilkiw JE. (2014) Assessment of burnout in veterinary medical students using the Maslach Burnout Inventory-Educational Survey: a survey during two semesters. BMC medical education.14:255.10.1186/s12909-014-0255-4

## 37) Analysis of massive transfusion protocols in six level 1 trauma centers in the Netherlands

### Tim W.H. Rijnhout^1^, Annemarije Bek^2^ , Femke Noorman, MD^3^, M.Zoodsma^3^, Rigo Hoencamp^4^

#### ^1^ Department of Surgery – section Traumasurgery, Radboud University Medical Center, Nijmegen, The Netherlands; ^2^ Dutch Defence Academy, Breda, The Netherlands; ^3^ Military Blood Bank, Ministry of Defense, Leiden, The Netherlands; ^4^ Ministry of Defense and Department of Surgery, Alrijne Medical Center Leiderdorp, Leiden University Medical Center, Leiden, The Netherlands

##### **Correspondence:** Tim W.H. Rijnhout (tim.rijnhout@radboudumc.nl)

**Background**

In hospital massive transfusion protocol (MTP) consist of administration of liquid stored blood components (erythrocytes/plasma/platelets) in equal ratio with additional medication. In mass casualty situations it is likely that equal ratios cannot be achieved because of insufficient components. To bridge this logistical threshold, the Dutch Ministry of Defence initialised the massive transfusion with frozen blood products (MAFOD) study. A randomized clinical multicenter trial in level 1 trauma centers that compares frozen with liquid stored platelets in the treatment of patients with major haemorrhage. In preparation of the MAFOD study the transfusion protocols of the participating centers were mapped.

**Aim**

The primary goal of this study is to assess the current MTP strategies in level-1 trauma centers in the Netherlands and compare these with current international literature.

**Methods**

Six level 1 trauma centers were invited to analysis their MTP. After a questionnaire was send, trauma surgeons and anaesthesiologists were asked to respond orally and send their current documented MTP.

**Results**

In the six participating centers the transfusion ratio is 3:3:1, 5:5:1 or 5:3:1. The amount of tranexamic acid administered was 2 grams in two centers and 1 gram in four centers. Fibrinogen was given in four centers based on clinical evaluation and in two centers guided by lab. Prothrombin complex administration was reported in four centers. Initiation of the MTP was clearly described, but information regarding termination was lacking.

**Conclusion**

Transfusion ratio and use of medication in MTPs differed between institutes and from international guidelines. The differences in transfusion ratio can be explained by (inter)national differences in type and volume of blood components and/or interpretation of the “1:1:1” guideline. A common protocol is preferably to be used in the participating centers of the MAFOD study and should be addressed in future research.

## 38) One year on following the remodeling of the Physician Response Unit “Taking the Emergency Department to the Patient”: an analysis of output and cost impact

### Lisa Ramage, Sophie Mitchinson, Tony Joy

#### Physician Response Unit, London’s Air Ambulance, Bart’s Health NHS Trust. Royal London Hospital, Stepney Way London E1 1BB

##### **Correspondence:** Tony Joy (Tony.Joy@bartshealth.nhs.uk)

**Introduction**

The Physician Response Unit (PRU) is an advanced emergency medical service responding to undifferentiated 999 calls across Northeast London. Operational since 2001[1], the service was remodeled in September 2017. Staffed by an emergency doctor and Emergency Ambulance Crew (EAC), it operates 365 days/year, aiming to ‘take the emergency department to the patient’. This observational study aims to explore the output of the PRU over its first year since remodeling and to estimate cost savings generated for the National Health Service (NHS).

**Methods**

Data was collected prospectively regarding each patient encounter. For those treated in the community, the treating team documented the agreed likelihood that the patient would have otherwise been a) conveyed to the emergency department (ED), and b) admitted to hospital. Health Episode Statistics (HES) data[2] and 2015/16 NHS referencing costs[3] were utilised to estimate cost savings generated.

**Results**

Over the 365-day period, the PRU treated 1924 patients. In 75% of cases, the PRU was the sole resource required, resulting in a minimum of 3 London Ambulance Service (LAS) resources/day being otherwise available. 33.3% of patients underwent advanced diagnostics not routinely available within the LAS. 67% were treated in the community. Of those not conveyed, it was agreed 84.2% would have otherwise been taken to an ED and 19.9% would subsequently have been admitted. An estimated 1085 ED attendances and 1326 bed days were avoided. Overall cost savings of £823,143 were generated.

**Discussion**

The PRU allows delivery of high quality emergency care, has proven successful in reduction of hospital conveyances and admissions, and has generated tangible cost savings to the NHS health economy. We believe this model to be capable of having similarly positive impact across a wider geographical area, and could be replicated across London to deliver significant benefit to patients, frontline staff and the wider NHS.

**References**

1. Bell A, Lockey D, Coats T, Moore F, Davies G. (2006) Physician Response Unit - a feasibility study of an initiative to enhance the delivery of pre-hospital emergency medical care. Resuscitation.69:389-93.10.1016/j.resuscitation.2005.10.013

2. Digital NHS. Hospital Episode Statistics [2017]. Available from: https://digital.nhs.uk/data-and-information/data-tools-and-services/data-services/hospital-episode-statistics.

3. Department of Health. NHS reference costs 2015/16 2016. Available from: https://www.gov.uk/government/publications/nhs-reference-costs-2015-to-2016.

## 39) Long term impacts of trauma on return to work and need for medical benefits

### Oddvar Uleberg^1^, Kristine Pape^2^, Thomas Kristiansen^3^, Pål R Romundstad^2^, Pål Klepstad^4^

#### ^1^Department of emergency medicine and pre-hospital services, St. Olav`s University Hospital, Trondheim, Norway; ^2^Department of public health, Faculty of medicine and health sciences, NTNU, Norwegian University of Science and Technology, Trondheim, Norway; ^3^Department of anaesthesiology, Division of emergencies and critical care, Oslo University Hospital, Rikshospitalet, Oslo, Norway; ^4^Department of anaesthesiology and intensive care medicine, St Olav’s University Hospital, Trondheim, Norway

##### **Correspondence:** Oddvar Uleberg (oddvar.uleberg@stolav.no)

**Background**

Long term functional outcomes are important as most patients survive their trauma. The objective of this study [1] was to describe the long-term consequences of trauma in patients with traumatic injury in a healthcare region.

**Materials and methods**

Trauma patients aged 16-65 years, active in work or education and admitted to hospitals in Central-Norway between 01.06.07 to 30.05.10 were included. Clinical data were linked to Norwegian national registries on cause of death, sickness and disability benefits, employment and education with a follow-up time until seventy-two months. Primary outcome measures were receipt of medical benefits and time to return to pre-injury work level. Secondary outcome measures were mortality within 30-days and mortality during the follow-up period.

**Results**

1191 patients were included of whom sixteen percent (n = 193) of the patients were severely injured (Injury Severity Score [ISS] > 15). Five years after injury the prevalence of medical benefits was 15.6 % among workers with minor injury, 22.3 % in moderately injured and 40.5 % in workers with severe injuries. Corresponding figures in students were; 9.1 % minor, 19.4 % moderate and 18.9 % severe, respectively. The median times after injury until return to work were 1, 4 and 11 months for patients with minor, moderate and severe injuries, respectively. Median time to return work in patients with and without severe head injury was 11 and 2 months, respectively. Twelve patients died within 30-days and an additional 17 (1.4 %) patients died during the follow-up period.

**Conclusions**

This study demonstrates that patients experiencing minor, moderate and major trauma initially received high levels of medical benefits; however, most recovered within the first year and resumed pre-injury work activity. Patients with severe trauma were more likely to receive medical benefits and have a delayed return to work.

**Reference**

1. Uleberg O, Pape K, Kristiansen T, Romundstad PR, Klepstad P. Population-based analysis of the impact of trauma on longer-term functional outcomes. Br J Surg. 2018 Sep 17. doi: 10.1002/bjs.10965.

## 40) Cycle helmet use is associated with reduced severe traumatic brain injury and death in a national study of 11285 cyclist major trauma patients.

### Nick Dodds^1^, Rowena Johnson^1^, Benjamin Walton^1^, Omar Bouamra^2^, David Yates^2^, Fiona Lecky^2^ and Julian Thompson^1^

#### ^1^ Severn Major Trauma Network, Bristol, UK; ^2^ Trauma Audit Research Network, University of Manchester, Salford, UK

##### **Correspondence:** Nick Dodds (nickdodds@nhs.net)

**Background**

Increased participation in cycling has been associated with an increase in UK cycling injuries with traumatic brain injury being the commonest cause of cycling death^1^. Controversy persists regarding the effectiveness of cycle helmet use with significant political and public health debate into this issue^2,3^. This study analysed a national database to assess the impact of helmet use on rates and pattern of injury in cyclists suffering from major trauma.

**Methods**

The NHS England Major Trauma Audit - the Trauma Audit and Research Network (TARN) database was interrogated to identify all adult (≥16 years) patients presenting to hospital with cycling-related major injuries, during a period from 14 March 2012 to 30 September 2017. TARN injury descriptors were then used to compare patterns of injury, care and mortality in helmeted versus non-helmeted cohorts.

**Results**

11285 patients presented with an injury due to a cycling accident during the study period. Data on the use of cycling helmets was available in 6621 patients. There was a significantly higher crude 30 day mortality in un-helmeted cyclists 5.6% (4.8% - 6.6%) versus helmeted cyclists 1.8% (1.4% - 2.2%) (p < 0.001). This persisted in a sensitivity analysis which assumed that all those with unknown cycle helmet status were not wearing a helmet. Cycle helmet use was also associated with a reduction in severe TBI (p < 0.001), ICU requirement (p<0.001) and neurosurgical intervention (p<0.001). There was a statistically significant increase in chest, spinal, upper and lower limb injury in the helmeted group in comparison to the un-helmeted group (all p<0.001).

**Discussion**

This national study demonstrates that severe traumatic brain injury and adjusted mortality is significantly reduced by the wearing of helmets in hospitalized injured cyclists.

**References**

1. Morgan AS, Dale HB, Lee WE, Edwards PJ. Deaths of cyclists in London: trends from 1992 to 2006. BMC Public Health. BioMed Central; 2010 Nov 15;10(1):1930.

2. https://www.cyclinguk.org/campaigning/views-and-briefings/cycle-helmets

3. https://www.rospa.com/rospaweb/docs/advice-services/road-safety/cyclists/cycle-helmets-factsheet.pdf

## 41) A systematic review of blood and CSF biomarkers as early prognosticators in severe traumatic brain injury

### William Seligman^1^, Nick Dodds^2^, Sebastian Bourn^4^, Andy Ray^2^, Skylar Paulich^2^, Marius Rehn^5^, Julian Thompson^2,3^

#### ^1^ Imperial School of Anaesthesia, London, UK; ^2^ Severn Major Trauma Network, Bristol, UK; ^3^ Great Western Air Ambulance, Bristol, UK; ^4^ Great North Air Ambulance Service, Darlington, UK; ^5^ Department of Research. Norwegian Air Ambulance Foundation. Oslo, Norway

##### **Correspondence:** William Seligman (seligmanw@gmail.com)

**Background**

TBI is devastating, for the individual patient, family members, and society at large [1]. Part of the emotional overlay of this is that individualised prognostication in TBI is difficult. As a result, many groups have analysed biomarkers with a view to improving prognostication [2]. Here, we have undertaken an initial analysis for a systematic review into biomarkers for acute severe TBI and present our initial findings.

**Methods**

This systematic review was registered with Prospero (CRD42018095632) and searched for prognosticating studies involving all biomarkers sampled acutely (within 48 hrs) from adult patients (>16 yrs) presenting with severe TBI (WFNS definition).

**Results**

Once duplicates were excluded, 1823 abstracts were included in this analysis. Of these, 265 studies met full text eligibility. Of these 178 studies, 45 studies were conference abstracts, and 132 were original abstracts (1 PhD thesis). 131 studies sampled blood, 59 studies involved sampling CSF. 13 studies involved microdialysis.

The most common biomarker sampled was S100B (37 studies), though in total 47 individual biomarkers for TBI were sampled (not including cytokines, markers of blood brain barrier dysfunction and markers of oxidative stress).

Mortality was an outcome measure in 65 studies. Morbidity was most commonly measured using Glasgow Outcome Score, though length of follow up different significantly: GOS at discharge (14 studies), 3 months (9), 6 months (41), 1 year (9).

**Discussion**

Full text review of 178 studies has shown significant heterogeneity in design, with differences in both biomarker sampling and outcome measures. Standardisation of future study protocols would enable comparison of putative biomarkers of TBI.

**References**

1.Murray CJ, Lopez AD. Global mortality, disability, and the contribution of risk factors: Global Burden of Disease Study. The Lancet. 1997 May;349(9063):1436–42.

2. Kochanek PM, Berger RP, Bayr H, Wagner AK, Jenkins LW, Clark RS. Biomarkers of primary and evolving damage in traumatic and ischemic brain injury: diagnosis, prognosis, probing mechanisms, and therapeutic decision making. Current Opinion in Critical Care. Current Opinion in Critical Care; 2008 Apr;14(2):135–41.

## 42) Out-of-hospital cardiac arrest following trauma: what does a Helicopter Emergency Medicine Service offer?

### E. ter Avest, J. Griggs, C. Prentice, J. Jeyanathan, R. M. Lyon

#### Air ambulance trust Kent, Surrey and Sussex, Redhill, Surrey, UK

##### **Correspondence:** E. ter Avest (EwoudterAvest@aakss.org.uk)

**Introduction**

Helicopter emergency medical services (HEMS) are often dispatched to patients in traumatic cardiac arrest (TCA) as they can provide treatments and advanced interventions in the pre-hospital environment that have the potential to contribute to an increased survival. This study, aimed to investigate the added value of HEMS in the treatment of TCA.

**Methods**

We performed a retrospective cohort study of all patients with a pre-hospital TCA who were attended by a non-urban HEMS (Air Ambulance trust Kent Surrey and Sussex) between July 1^st^ 2013 and May 1^st^ 2018. We investigated how many patients got return of spontaneous circulation (ROSC) at scene, which HEMS specific advanced interventions were performed in these patients, and how these interventions were related to ROSC.

**Results**

During the study period 263 patients with a TCA were attended by HEMS with an average response time of 30 minutes [range 13-109]. 51 patients (20%) regained ROSC at scene (28 before- and 23 after arrival of HEMS). The HEMS specific interventions of blood product administration (OR 8.54 [2.84-25.72]), and RSI (2.95 [1.32-6.58]) were positively associated with ROSC. Most patients who had a ROSC had one or more HEMS specific interventions being performed - RSI (n=19, 37%), blood product administration (n=32, 62%), thoracostomies (n=36, 71%) and thoracotomy (n=1, 2%). HEMS also delivered other important interventions to these patients as IV/IO access (n=20, 39.2%) and endotracheal intubation without drugs (n=9, 17.6%).

**Conclusion**

HEMS teams should be involved in the treatment of patients with a TCA, even in non-urban areas with prolonged response times, as they provide knowledge and skills that contribute to regaining and maintaining a sustained ROSC in this critically ill and injured cohort of patients.

## 43) Prehospital lactate in trauma patients: What are we measuring?

### Ewoud ter Avest, Joanne Griggs, Julian Wijesuriya, Malcolm Russell, Richard Lyon

#### Air ambulance trust Kent, Surrey and Sussex, Redhill, Surrey, UK

##### **Correspondence:** Ewoud ter Avest (EwoudterAvest@aakss.org.uk)

**Introduction**

Trauma triage and risk stratification is a challenging component of pre-hospital emergency medicine. Point of care serum lactate measurement is emerging as an adjunct to pre-hospital clinical assessment and has the potential to guide triage and advanced treatment decision-making [1-3]. However, factors influencing pre-hospital lactate and the influence it has on clinical decision-making and pre-hospital care remain poorly understood.

**Method**

A retrospective analysis of trauma patients attended by the Air Ambulance, Kent, Surrey & Sussex (AAKSS) between 13 July 2017 and 24 April 2018 in whom a pre-hospital lactate was measured. The clinical endpoints of interest were the association of various patient and treatment characteristics with absolute and elevated pre-hospital lactate levels.

**Results**

During the study period, lactate was measured in 156 trauma patients. Median lactate was 3.0 mmol/l. Patients with an elevated lactate more often had head injuries (62% vs 41%, *p* = 0.008), and deranged indices of end organ perfusion- (shock index 0.80 [0.58-1.03] vs 0.61 [0.40-0.82], *p* < 0.001) and oxygenation (SpO_2_ 96[89-100]% vs 98 [96-100%], *p* = 0.025), whilst IV analgesia was administered less often (51.6% vs 67.2%, *p* = 0.03). In multivariate analysis, indices of hypoperfusion and oxygenation only explained 15% of the variation in lactate levels.

**Conclusion**

The etiology of elevated lactate levels in trauma patients is multifactorial and cannot be assumed to be solely the result of end-organ hypoperfusion. Clinicians should therefore be cautious about basing advanced treatment decisions on pre-hospital lactate measurements and further research is warranted.

**References**

1. Lewis CT, Naumann DN, Crombie N, Midwinter MJ. Prehospital point-of-care lactate following trauma: A systematic review. J Trauma Acute Care Surg. 2016 ;81:748-55.

2. St John AE, McCoy AM, Moyes AG, Guyette FX, Bulger EM, Sayre MR. Prehospital Lactate Predicts Need for Resuscitative Care in Non-hypotensive Trauma Patients. West J Emerg Med. 2018;19:224-31.

3. Guyette FX, Meier EN, Newgard C, McKnight B, Daya M, Bulger EM, et al. A comparison of prehospital lactate and systolic blood pressure for predicting the need for resuscitative care in trauma transported by ground. J Trauma Acute Care Surg. 2015;78:600-606.

## 44) Prehospital emergency anaesthesia in paediatric patients: the experience of physician-led air ambulance service

### Charlotte Lindsay^1^, Jonathan Dean^2^, Drew Welch^3^

#### ^1^ Department of Emergency Medicine, Norfolk and Norwich University Hospitals NHS Foundation Trust, Norwich, UK; ^2^ Department of Anaesthesia, Norfolk and Norwich University Hospitals NHS Foundation Trust, Norwich, UK; ^3^ East Anglian Air Ambulance, Norwich, UK

##### **Correspondence:** Charlotte Lindsay (charlotte.lindsay@nhs.net)

**Introduction**

Pre-Hospital Emergency Anaesthesia (PHEA) in paediatric patients is a relatively uncommon procedure performed by prehospital doctors, many of whom have limited experience of paediatric anaesthesia.^1^

**Methods**

A retrospective observational study of all paediatric trauma patients attended by the East Anglian Air Ambulance between February 2015 and July 2018 was conducted. Cases where PHEA was administered were examined to evaluate the quality and safety of the procedure. Appropriate drug doses were calculated based on age, weight and haemodynamic stability of the patient in accordance with the standard operating procedure for PHEA. Appropriate observations for age were calculated using the service’s paediatric aide memoire. Intubations occurring without the use of drugs or for a medical reason were excluded.

**Results**

We identified 254 paediatric trauma patients, 17 (6.7%) of which underwent PHEA. In all cases the primary indication was low GCS. The most common mechanisms of injury were road traffic collisions (76.4%), falls (17.6%) and penetrating injury (5.8%). Fourteen (82.4%) patients received appropriate doses of fentanyl, ketamine and rocuronium for induction. All anaesthetics were maintained with midazolam, ketamine or propofol with only 7 (58.8%) patients given sufficient dosing. Complications were common with 12 (70.5%) anaesthetics complicated by desaturation, bradycardia or hypotension and 82.4% complicated by hypercapnia. One brief episode of cardiac arrest occurred on induction of an unstable patient.

**Conclusion**

Pre-hospital emergency anaesthesia of the critically injured child is a high-risk procedure with frequent complications. The risks and benefits of PHEA should be carefully considered before performing this with limited paediatric anaesthetic experience. Additional training and education is vital to develop and maintain competency in this rarely performed but critical intervention.

**Reference**

1. Lockey, D.J., Crewdson K, Davies G et al. AAGBI: Safer pre-hospital anaesthesia 2017. Anaesthesia. 2017 Mar;72(3):379-390.

## 45) Trauma and the obesity epidemic: A systematic review of the impact of obesity on trauma mortality and morbidity.

### Simon Mayer

#### Centre for Trauma and Neuroscience, Blizard Institute – Queen Mary University of London

##### **Correspondence:** Simon Mayer (simon_mayer@hotmail.co.uk)

**Background**

Trauma and obesity are both current global epidemics. A simple way to measure the body habitus of patients, to identify the overweight or obese is via the internationally recognized calculation of body mass index (BMI). The primary aim of this systematic review is to assess the mortality rate of those patients with a BMI > 30kg/m^2^ in relation to traumatic injury and secondly to assess the effect of those patients with BMI > 30kg/m^2^ upon the length of stay in hospital with regards to traumatic injury.

**Method**

A systematic review of the literature was conducted via an internet search of databases and hand searching of references in identified publications from 1^st^ January 1990 to 17^th^ February 2018. Data was extracted from identified publications to include odds ratios of mortality and total length of stay in hospital (days) for patients with a BMI >30kg/m^2^ from included studies when compared to patients with a BMI <24.9kg/m^2^.

**Results**

A total of 23 studies met the inclusion criteria, 32, 378 patients being admitted to hospital with a BMI >30kg/m^2^ and recorded ISS. Datum obtained identifies injury severity scores (ISS) 19.93 vs 22.3 for obese versus non-obese respectively. Data collated identifies obese patients OR. 1.66 (95% CI 0.75 – 4.2) greater to suffer mortality than those normal-weight BMI <24.9 kg/m^2^. Furthermore, those categorised as obese have 3.78 additional days in the hospital compared to those defined as normal weight.

**Conclusion**

This systematic review presents a strong association of increased mortality in trauma patients with a BMI >30kg/m^2^ when compared to non-obese, despite having a reduced ISS. However, the direct relationship between obesity and mortality is not fully understood at present. Evidence suggests those who have a BMI >30kg/m^2^ are more likely to suffer detrimental effects following trauma due to preexisting co morbidities.

## 46) Mode of death and organ donation referral rate following hospital admission from out-of-hospital cardiac arrest in a tertiary cardiac centre

### Johnny Hall, Sian A Moxham, Matt Thomas

#### Critical Care, University Hospitals Bristol NHS Foundation Trust, Bristol Royal Infirmary, Upper Maudlin Street, Bristol, UK

##### **Correspondence:** Matt Thomas (matt.thomas@uhbristol.nhs.uk)

**Background**

50,000 people suffer an out-of-hospital cardiac arrest (OOHCA) per annum in the United Kingdom (U.K.); 10% of patients survive to hospital discharge. Two-thirds of patients, who do not survive to hospital discharge, die from neurological injury with one third of deaths attributable to cardiac dysfunction[1].

Death from cardiac dysfunction is typically early (<72hours) compared to death from neurological injury (>72 hours)[2]. Accurate neuroprognostication should occur at least 72 hours post return of spontaneous circulation (ROSC)[3].

The objective of this study was to determine the mode of death of all non-survivors of an OOHCA in a single tertiary cardiac centre.

**Method**

This was a retrospective audit of a 21-bedded critical care unit at a tertiary cardiac centre with 24/7 cardiac catheter laboratory access. The study population was generated from ICNARC data. All patients who died post OOHCA between January 2017 and May 2018 were eligible for analysis. 78 patients were identified but 1 patients data could not be accessed by the system, leaving 77 patients data for analysis. Data was retrospectively collected from Philips CIS System database, ICE system and Evolve. Analysis was conducted using Microsoft Excel and IBM SPSS.

**Results**

74% (57/77) patients died, or had life support withdrawn, due to devastating neurological injury. 13% (10/77) died from cardiogenic dysfunction. 13% (10/77) had a mixed picture or died from other modes e.g. ischaemic bowel, pulmonary embolism, sepsis. 95% (73/77) were referred to the organ donation service.

**Discussion**

The predominant mode of death, as previous studies have shown, is neurological injury. However, the proportion is higher with a likely reduction in patients dying from cardiogenic shock. This raises the possibility that concentrating critical care expertise, with easy and early access to cardiological support, increases the likelihood of survival from cardiogenic shock to either neurological survival or neurological death.

**References**

1. Nolan JP, Laver SR, Welch CA, Harrison DA, Gupta V, Rowan K. Outcome following admission to UK intensive care units after cardiac arrest: a secondary analysis of the ICNARC Case Mix Programme Database. Anaesthesia 2007; 62: 1207-1216.

2. Laurent I, Monchi M, Chiche JD, Joly LM, Spaulding C, Bourgeois B, et al. Reversible myocardial dysfunction in survivors of out-of-hospital cardiac arrest. Journal of the American College of Cardiology 2002; 40: 2110-2116.

3. Samaniego EA, Mlynash M, Caulfied AF, Eyngorn I, Wijman CA. Sedation confounds outcome prediction in cardiac arrest survivors treated with hypothermia. Neurocritical Care 2011; 15: 113-119.

## 47) Emergency Department Airway Management in a DGH

### Owen Lewis^1^, Edward Valentine^2^

#### ^1^ Anaesthetic Department, University Hospital of Wales, Cardiff, Wales, UK; ^2^ Emergency Department, Royal Gwent Hospital, Newport, Wales, UK

##### **Correspondence:** Owen Lewis (o.lewis24@gmail.com)

**Background**

The Royal College of Anaesthetists (RCoA) 4^th^ national audit project [1] identified the Emergency Department (ED) as a high-risk area for provision of emergency airway management. Accordingly the RCoA and Royal College of Emergency Medicine have published guidance [2,3] recommending regular audit of ED airway management, with specific recommendations around seniority of staff, complications and documentation. This project aimed to audit a total of 50 ED rapid sequence inductions, identifying problems and subsequently making improvements to practice.

**Methods**

An RSI audit form was designed using the minimum data collection and key performance indicators outlined in the AAGBI safer pre hospital anaesthesia guideline. Staff awareness of the project was achieved via posters in the ED, ED nursing staff briefings and e-mails to all trainee anaesthetists. Data collection began in July 2016 and a total of 47 cases reached by April 2017.

**Results**

In all cases the most senior person present was at the level of ST3 or above. In 49% of cases an Emergency Physician was the most senior person present and an Anaesthetist or Intensivist in 40% and 11% respectively. Regarding complications, in 18% of cases the lowest SpO2 recording was <92% and in 20% of cases the lowest systolic BP recording was <90mmhg. There were no cases of failed intubation.

**Discussion**

Overall our data suggests good compliance with national standards regarding airway management in RGH ED. However, we believe many RSI’s performed were not captured, reducing the confidence we can have in this finding. To address this, we are introducing an ‘Emergency Anaesthesia Record’ aiming to stop duplication of work, improve documentation, data capture and more accurately pick-up complications such as hypoxaemia and hypotension. We are implementing a similar chart in a neighbouring teaching hospital, creating a large database and a change in culture across the Welsh Deanery.

**References**

1. T. M. Cook, N. Woodall, C. Frerk, on behalf of the Fourth National Audit Project; Major complications of airway management in the UK: results of the Fourth National Audit Project of the Royal College of Anaesthetists and the Difficult Airway Society. Part 1: Anaesthesia, *BJA: British Journal of Anaesthesia*, Volume 106, Issue 5, 1 May 2011, Pages 617–631, 10.1093/bja/aer058

2. D Turnbull, H Krovvidi, J Gannon, Guidelines for the Provision of Anaesthesia Services in the Non-theatre environment 2018. Available at https://www.rcoa.ac.uk/document-store/guidelines-the-provision-of-anaesthesia-services-the-non-theatre-environment-2018 Last accessed 30/10/18.

3. Emergency airway management: a joint position statement from the Royal College of Emergency Medicine and the Royal College of Anaesthetists. RCoA and RCEM, 2015 Available at https://www.rcoa.ac.uk/sites/default/files/RCEM-RCoAEmAirMan2015.pdf Last accessed 30/10/18

## 48) Traumatic Damage Control Surgery – Surgical Pause Form

### Clementine Stubbs, Matthew Wyse, Laura May

#### Department of Anaesthetics, University Hospitals Coventry and Warwickshire, Coventry, UK

##### **Correspondence:** Clementine Stubbs (clementine.stubbs@nhs.net)

**Background**

Prolonged operative time and persistent bleeding put major trauma patients at risk of a ‘lethal triad’ of coagulopathy, acidosis and hypothermia. Damage Control Surgery (DCS) is a concept of truncated surgery that aims to restore normal physiology as a priority to restoring normal anatomy.[1] DCS is facilitated by a multidisciplinary team in a high-pressured, time-critical environment. A high level of situational awareness in the whole team is required to maintain focus on minimising the surgical and physiological stress and maximising resuscitation.[2]

**Method**

The Surgical Pause Form recently introduced at University Hospital Coventry facilitates shared clinical understanding for effective management of DCS. The A1 chart displayed in theatre identifies the leads for resuscitation, anaesthetics and surgery. The patients’ injuries, surgical plan and important physiological parameters including temperature and blood gas analysis are recorded. There are prompts to implement the TRAUAMTIC principles for effective control of major haemorrhage.[3] The clock starts at time of surgery and pauses are enforced every thirty minutes to review current clinical condition, transfusion status and surgical progress, and to discuss what DCS is still required, if any. In order to gauge how DCS is conducted in other Major Trauma Centres (MTCs) a three-question survey was sent to all MTC leads in England.

**Results**

Eight MTCs responded, three of which follow a DCS protocol using a documentation aid and mandatory surgical pauses. One MTC reported using surgical pauses only. Some MTCs without protocolised DCS expressed an interest in the use of our documentation aid which, locally has been well regarded by all specialties involved.

**Conclusion**

Our DCS form, as a cognitive aid, is applicable for use in all settings where DCS is carried out. There may be particular benefit in settings with lower case volumes such as trauma units where DCS principles are less well embedded.

**References**

1. Jaunoo S et al. Damage control surgery. International Journal of Surgery. 2009; 7(2):110-113.

2. McCullough A, Haycock J, Forward D, Moran C. Early management of the severely injured major trauma patient. British Journal of Anaesthesia. 2014; 113(2):234-241.

3. May L, Kelly A, Pipe J. Traumatic: an evidence-based reference tool for coordinated, consistent and optimal management of major haemorrhage and major trauma. Anaesthesia News. 2016; 361:15.

## 49) Civilian outcomes in international terror attacks: a systematic review

### Sophia E Lewis^1 2^, Daniel C Worley^2^, Benjamin F Rosen^3^

#### ^1^Imperial College, London UK; ^2^Essex Bedfordshire and Hertfordshire Foundation School, UK; ^3^North Central and East London Foundation School, London UK

##### **Correspondence:** Sophia E Lewis (sophiaelewis@doctors.org.uk)

**Background**

Terrorism is a globally evolving phenomenon, worldwide incidence has risen from 651 in 1970 to 10,900 in 2017 [1], and continues to spread, with two thirds of all countries experiencing terrorism in 2016 [2]. Despite the threat to life and healthcare systems, the published evidence base is limited, and no systematic reviews are available.

**Method**

This review aimed to explore the effect of international terror on non-combatants for the preceding 50 years (1968-2018). Using MEDLINE, Embase, Global Health and the Cochrane library databases, we investigated the outcomes of overall mortality, case fatality ratio, critical mortality and critical injury. The main factors investigated were: economic rank of the host nation, physical injuries, and mechanism of the event.

**Results**

Of 886 articles reviewed, 114 met final inclusion criteria. We identified over 23000 casualties of terrorism and 4000 fatalities. Due to variable datasets within the papers, only two factors were used for meta-analysis. For LMICs there was a more than two-fold increase in risk of mortality in case fatality ratio (OR 2.69 CI 2.63-2.77) and an increase in critical mortality (OR 1.68 CI 1.53-1.83). There is a two-fold increase in case fatality ratio in terrorist shootings compared to other modalities (OR 2.50 CI 2.37-2.59). Small trend analysis was done to investigate other factors.

**Conclusion**

Terrorism is disproportionately lethal in LMICs, likely due to limited healthcare resources and a lack of formal pre-hospital care. With currently available data it is not possible to find the main factor that would affect mortality, although terrorist shootings appear to be the most lethal mechanism. This review highlights the need for more systematic and comparable data worldwide to further investigate this topic.

**References**

[1] Global Terrorism Database [Internet] National Consortium for the Study of Terrorism and Responses to Terrorism [Updated July 2018, cited 04 Nov 2018] Available from: http://apps.start.umd.edu/gtd/

[2] Global Terrorism Index [Internet] Institute for Economics and Peace [Updated 2017, cited 04 Nov 2018] Available from: http://globalterrorismindex.org

## 50) Is thoracolumbar spine imaging required in all trauma patients who present after a fall from less than 2 metres? A retrospective analysis of 3252 patients.

### Hazel M Luck^1^, Jules Brown^2^, Timothy Hooper^2^, Julian Thompson^3^

#### ^1^Bristol Royal Infirmary, Severn Deanery, Bristol, UK; ^2^ Intensive Care Unit, Southmead hospital, North Bristol NHS Trust, Bristol, UK; ^3^Severn Major Trauma Network, Bristol, UK

##### **Correspondence:** Hazel M Luck (hazel_luck@hotmail.co.uk)

**Background**

Evidence suggests that there is a high frequency of significant injury with a mechanism of fall from standing height [1]. There is also evidence to suggest that clinical examination is poor [2] and that imaging is required to diagnose spinal fractures [3]. The aim of our retrospective study was to ascertain the incidence of thoracolumbar injuries in those patients who have a fall of less than 2 metres and determine whether groups could be risk stratified to aid decision-making regarding the need for thoracolumbar imaging.

**Method**

A retrospective observational study of trauma patients who fell less than 2 metres and were admitted to a single Major Trauma Centre in the Southwest of the United Kingdom April 2012-March 2017. The National Trauma Audit and Research Network (TARN) database data was used. Patient demographics, vital signs, imaging undertaken, injuries sustained, in hospital course and patient outcome were all included for analysis using pivot tables.

**Results**

53% (3252) of patients recorded on the TARN database had a mechanism of fall <2m. Of these, 10.9% (353) patients sustained thoracic or lumbar injuries (TL injuries). No single group had a low/minimal incidence when analysed by age, sex, GCS, requirement for mechanical ventilation or ISS score. 11.2% of those ≤65 years old and 9.9% of those >65 years old sustained TL fractures.

**Discussion**

This study aimed to identify those who were at high risk of TL injuries who would require automatic imaging of thoracic and lumbar spine and those in whom the incidence was low enough that they could safely avoid imaging. We did not identify any low risk groups and found that the incidence across all groups was around 10%. This data supports routine thoracolumbar imaging of major trauma patients who present to hospital after falling from standing.

**References**

1. Hao W, Coppola M, Robinson R, Scribner J, Vithalani V, de Moor C, Gandhi R, Burton M, Delaney K. Geriatric Trauma Patients With Cervical Spine Fractures due to Ground Level Fall: Five Years Experience in a Level One Trauma Center. Journal of clinical medicine research. 2013; Apr;5(2):75-83.

2. Inaba K, Nosanov L, Menaker J, et al. Prospective derivation of a clinical decision rule for thoracolumbar spine evaluation after blunt trauma. J Trauma Acute Care Surg. 2015; 78:459-467.

3. Riddell, J Inaba K, Jhun P, Herbert M. A Clinical Decision Rule for Thoracolumbar Spine Imaging in Blunt Trauma? Annals of Emergency Medicine. 2016; 68(6): 781 - 783.

## 51) Evaluating the impact of prehospital blood transfusion

### Olivia V Cheetham^1^, Emma J Shepherd^1^, Matthew JC Thomas^1^, Harvey Pynn^1^, Timothy Hooper^1^, Julian Thompson^2^, Patrick Morgan^1^

#### ^1^Great Western Air Ambulance Charity, South West Ambulance Service Trust, UK; ^2^Severn Major Trauma Network, Bristol, UK

##### **Correspondence:** Olivia V Cheetham (oliviacheetham@googlemail.com)

**Background**

Early blood product transfusion following traumatic massive haemorrhage has been associated with increased survival [1]. Prehospital transfusion protocols are increasingly being used, but there is limited published data on suitable transfusion triggers and clinical impact of this innovation [2,3]. This study examined the case selection and physiological impact following the introduction of a warmed 2 unit packed red cell (PRC) transfusion protocol for the treatment of shocked major trauma patients with suspected ongoing life-threatening haemorrhage attended by Pre Hospital Critical Care teams.

**Methods**

Retrospective review of pre and in-hospital documentation for trauma patients conveyed to a Major Trauma Centre (MTC) transfused by Great Western and Wiltshire Air Ambulance Teams between 08/2015 and 06/2017.

**Results**

Of 94 patients who received prehospital (PH) PRCs, 53 were major trauma patients transferred to the study MTC (median age 39.5yrs [IQR 27.6-58.3], 79% male, 83% blunt trauma). On MTC arrival, 95.2% (40/42) had pH <7.35, 90.2% (37/41) had base excess <-2.0 and 84.1% (37/44) had lactate >2.0. 83% received on-going transfusion (>2 units of blood products). N=46 had mean Injury Severity Score 40 [SD 18.3]; 23.9% died.

Paired initial PH and MTC observations were analysed. Statistically significant improvement in SBP (median PH 77mmHg[IQR 65-92mmHg], MTC 99mmHg[IQR 86-117mmHg]) and shock index (median PH 1.62[IQR 1.22-2.16], MTC median 1.16[IQR 0.89-1.38]) occurred following prehospital PRC transfusion (p<0.001). There was no significant difference in mean HR (PH 118bpm[SD 32.0], MTC 113bpm[SD 29.9], p=0.315^B^) or temperature (PH 35.6^O^C[SD 1.17], MTC 35.5 ^O^C[SD 1.03], p=0.647).

**Conclusions**

An appropriate patient cohort with significant ongoing transfusion requirements and high mortality was successfully identified and transfused early. Despite suspected ongoing bleeding, a warmed 2 unit prehospital transfusion demonstrated improved ongoing haemodynamic instability and was not associated with worsening hypothermia. This service innovation demonstrates the feasibility of implementing advanced resuscitation in the prehospital setting.

**References**

1. MacLeod JB, Lynn M, McKenney MG, Cohn SM and Murtha M. Early coagulopathy predicts mortality in trauma. Journal of Trauma 2003; 55:39-44.

2. Weaver AE, Hunter-Dunn C, Lyon RM, Lockey D and Krogh CL. The effectiveness of a ‘Code Red’ transfusion request policy initiated by pre-hospital physicians. Injury 2016; 47(1): 3-6.

3. Rehn M, WeaverAE, Eshelby S, Røislien J and Lockey DJ. Pre-hospital transfusion of red blood cells in civilian trauma patients. Transfusion Med 2018; 28: 277-283.

## 52) Improving tube thoracostomy complication rates at a UK Major Trauma Centre over a five-year period

### Peter Sykes, Lauren Bose, Nicola Morgan

#### Emergency Department, Southmead Hospital, North Bristol NHS Trust, Bristol, UK

##### **Correspondence:** Peter Sykes (peter.sykes1@icloud.com)

**Background**

Tube thoracostomy (TT) is a potentially life-saving procedure in the context of major trauma [1]. Alarmingly, associated complication rates of 30% are reported [2]. Research suggests improving training is key in reducing iatrogenic harm [3]. In 2012, North Bristol Trust (NBT) became a Major Trauma Centre (MTC) and promptly recognised numerous complications following TT. This led to an education focused quality improvement project (QIP), which has achieved a sustained reduction in TT complications over five years.

**Method**

A retrospective audit was conducted for trauma patients who underwent TT from April 2012 to March 2013. Patients were identified using the Trauma Audit Research Network database and complications ascertained via inpatient notes and radiology. An educational programme was implemented at NBT providing regular teaching sessions from 2013. For trauma team doctors, teaching involved a lecture, small-group tutorial and practical skills session using manikins. Emergency Department nursing staff participated in small group workshops focused on chest drain equipment, assisting and principles of human factors and graded assertiveness. Re-audits one year (May 2013-July 2014) and five years post-intervention (January 2017- December 2017) were completed.

**Results**

80 chest drains were placed between April 2012 and March 2013. 15 (18.75%) were placed in lung parenchyma and 32 (40%) were malpositioned requiring manipulation. Post-intervention, one-year re-audit found 2/81 (3%) of drains were placed in lung parenchyma and 21/81 (26%) malpositioned. Five-year re-audit noted 5/82 (6.1%) of drains were parenchymal and 13/82 (15.9%) malpositioned.

**Conclusion**

This QIP achieved a sustained reduction in TT complications at NBT. These results perhaps reflect a change in trauma management toward a more conservative approach, using imaging modalities more frequently, which will positively impact patient safety. Nevertheless, our study also illustrates how a regular audit process and teaching programme, easily adoptable by other MTCs, can potentially produce substantial improvements in trauma care.

**Acknowledgements**

The authors would like to thank Dr. Alok Arora (Internal Medicine Specialist, Bay Area Medical Center, Wisconsin, USA), Dr. Ian Thomas (Consultant in Anaesthesia and Critical Care, Southmead Hospital, Bristol, UK) and Lt. Col. Timothy Hooper (Consultant in Anaesthesia and Critical Care, Southmead Hospital, Bristol, UK) for their significant contributions to this QIP.

**References**

1. Aylwin CJ, Brochi K, Davies GD, Walsh MS. Pre-hospital and in-hospital thoracostomy: indications and complications. Ann R Coll Surg Engl. 2008; 90(1): 54-7

2. Bailey RC. Complications of tube thoracostomy in trauma. J Accid Emerg Med. 2000; 17(2): 111-4

3. Ball CG, Lord J, Laupland KB, Gmora S, Mulloy RH, Ng AK, Schieman C, Kirkpatrick AW. Chest tube complications: how well are we training our residents? Can J Surg. 2007; 50(6): 450-8

## 53) A case report of ventricular fibrillation arrest in traumatic brain injury

### Ali J Watts^1^, Erica Ley^1^, Holly Clarke^2^, Ben Nimmo^3^

#### ^1^Essex and Herts Air Ambulance Trust, UK; ^2^Bart’s and The London Medical School, London. UK; ^3^University of Glasgow Medical School, Glasgow, Scotland

##### **Correspondence:** Ali J Watts (ali.watts@ehaat.org)

**Background**

We present a case of an 18 year old male motorcyclist involved in a road traffic collision with a van, who sustained a traumatic brain injury. Though initially with output, shortly after the incident he was found to be in ventricular fibrillation (VF). VF as the presenting rhythm in a traumatic cardiac arrest is rare as is association with TBI. We postulate potential causes for this based on literature review.

**Case Report**

A motorcyclist had a head-on high speed collision with a van causing a large dent to the bonnet and his helmet to come off. Bystander CPR was started on recognition of cardiac arrest and initial rhythm check identified VF that was successfully defibrillated with one 200J shock. Suspected injuries included an isolated head injury with CT confirmation of extensive subarachnoid haemorrhage, occipital and sphenoid cranial fractures. There was alleged cocaine and marijuana use and further cardiac investigation excluded any cardiac pathology.

**Conclusions**

Deasey et al. (2012) describe a prevalence of VF associated with traumatic arrests at 1.6%, this equates to 35 cases in 2187 traumatic cardiac arrests [1].

Possible causes of VF in traumatic arrest are: commotio cordis, subarachnoid haemorrhage and catecholamine surge following brainstem insult. In the acute phase subarachnoid haemorrhage as a cause of VF is less implicated [2] with this complication usually presenting later. VF arrest after trauma may be made more likely in the context of sympathomimetic drug use, pre-existing ischaemic heart disease and paediatric or adolescent populations [3]. Cocaine inhibits the reuptake of noradrenaline leading to excessive catecholamine action. This in combination with a catecholamine storm at the time of head injury may have precipitated the VF.

VF as the presenting rhythm in traumatic cardiac arrest is not common. Specific factors that increase the likelihood include traumatic brain injury and sympathomimetic drugs.

**References**

1. Deasy C, Bray J, Smith K, Harriss L, Morrison C, Bernard S. Traumatic out-of-hospital cardiac arrests in Melbourne, Australia. Resuscitation. 2012;83(4):465–70.

2. Inamasu J, Nakagawa Y, Kuramae T, Nakatsukasa M, Miyatake S. Subarachnoid Hemorrhage Causing Cardiopulmonary Arrest: Resuscitation Profiles and Outcomes. Neurol Med Chir. 2011; 51(9):619–23.

3. Mogayzel C, Quan L, Graves JR, Tiedeman D, Fahrenbruch C, Herndon P. Out-of-Hospital Ventricular Fibrillation in Children and Adolescents: Causes and Outcomes. Ann Emerg Med 1995;25(4):484–91.

## 54) Major Incident Trauma Simulation: A Novel Multiple Casualty Course

### Sophie Williams^1,2^, Kunal Babla^2,3^, Thomas Waring^2^, Simon Calvert^2,4^, Duncan Bew^1,2^

#### ^1^Department of Surgery, King’s College Hospital, London, UK; ^2^Department of Medical Education, King’s College Hospital, London, UK; ^3^Department of Paediatrics, King’s College Hospital, London, UK; ^4^Department of Emergency Medicine, King’s College Hospital, London, UK

##### **Correspondence:** Sophie Williams (sophie.williams15@nhs.net)

**Background**

UK Major Incidents (MI) and rate of trauma admissions are increasing. Though trauma simulation courses are available, access to multi-disciplinary, multiple casualty trauma simulation is limited. Our Major Trauma Centre (MTC) does not currently offer MI training for clinical staff. We present a novel, multiple casualty and high fidelity, Major Incident Trauma Simulation (MITS).

**Materials and Method**

Trauma scenarios were developed based on recent UK MIs. A dedicated, 7 bedded simulation room represented the resuscitation room. All trauma team members (TTMs) were invited to attend. The day comprised of a 90-minute, real-time simulation with high fidelity manikins, followed by a debrief session and afternoon lectures on topical themes. Debrief minutes were kept and feedback collected.

**Results**

24 TTMs attended. 21/24 completed feedback. 43% had previous MI experience, 57% had no experience. Pre-course, the majority of candidates felt not at all or slightly prepared (62%) for an MI, post-course, candidates felt better prepared, with 95% rating themselves as moderately or quite prepared. Confidence in candidate role during MIs also increased, with 52% feeling slightly prepared pre-course compared with 95% feeling moderately or quite prepared post-course. 100% found the cases relevant to their practice, with 100% wishing to participate in future courses. Common learning points included gaining familiarity with MI command structure, the importance of ‘sanitising’ potential assailants and resource management including rationing CT and theatre space.

**Conclusions**

In a climate of increasing MIs and UK terror threat status at severe, there is a need for preparedness amongst clinical staff. This model is an effective way of exposing TTMs to MIs in a protected setting. In addition, it represents a transferrable educational tool for multiple patient teaching in varied settings such as ward or recovery based.

## 55) How is laparoscopy utilised in United Kingdom trauma care for abdominal injuries?

### Kate Hancorn ^1^, Tom Stacey ^2^, Ashok Menon ^3^

#### ^1^ The University of Swansea, Swansea, Wales, UK; ^2^ The Trauma Audit and Research Network, University of Manchester, Manchester, UK; ^3^ Wirral University Teaching Hospital NHS Foundation Trust, Birkenhead, UK

##### **Correspondence:** Kate Hancorn (Kate.Hancorn@bartshealth.nhs.uk)

**Background**

Diagnosis of the injuries resulting from abdominal trauma presents a great challenge, even to the most experienced of trauma surgeons. The role of laparoscopy in trauma is still under debate. The rate of negative laparotomy in trauma has been noted to be up to 20% and despite improvements in imaging technologies has not decreased further.

**Aim**

To evaluate the current use of laparoscopy in patients presenting with abdominal trauma in the UK.

**Method**

An observational study of current practice in the UK was conducted using data available through the Trauma Audit and Research Network.

**Results**

507 patients underwent laparoscopy over the 12 year study period representing a conversion rate to open procedure of 43.8% (222/507), a negative laparotomy rate of 5% (11/222), non-therapeutic laparotomy rate of 18% (40/222) and missed injury rate of 3.5% (10/285). Laparoscopic sensitivity and specificity were calculated at 96.5% and 100% respectively. The rate of therapeutic laparoscopy for patients with identified injuries was 48.8% (105/215). The most common site of therapy being the small bowel and diaphragm.

**Conclusion**

This study is the only study to focus on the role of laparoscopy in abdominal trauma in the UK to date. It is evident laparoscopy is being performed safely for both penetrating and blunt injuries in a diagnostic and therapeutic capacity. Trauma laparoscopy remains an area for future study and further developments within clinical practice.

## 56) Extending post-PHEA observation analysis to 10 minutes reveals that Fentanyl/Ketamine induction protocols are associated with new hypotension in Major Trauma and non Major Trauma patients

### Victoria Hughes^1^, Johannes von Vopelius-Feldt^1^, Ed Valentine^1^, Julian Thompson^1,2^

#### ^1^Great Western Air Ambulance, Bristol, UK; ^2^Severn Major Trauma Network, Bristol, UK

##### **Correspondence:** Victoria Hughes (Vicki.hughes@nbt.nhs.uk)

**Background**

Variation exists in Pre Hospital Emergency Anaesthesia (PHEA) induction drug regimens^1^. Protocolised weight and cardiovascular stability adjusted ratios of fentanyl and ketamine have been demonstrated to reduce immediate post induction hypotension in major trauma patients^2^. This study assessed whether cardiovascular stability using this protocol was maintained for up to 10min post PHEA in major trauma patients and whether the protocol could be safely used in non-trauma critical care patients.

**Methods**

A retrospective analysis was performed of all GWAAC cases using the fentanyl/ ketamine PHEA protocol between 01/2017-08/2018. Patient age, estimated weight, pre-induction and lowest systolic blood pressure (SBP) within 10min of PHEA, pathology and induction drugs were analysed. Linear regression was used to examine the association between drug doses and lowest post-induction SBP.

**Results**

240 patients underwent PHEA using the fentanyl/ ketamine protocol during the study period. Within 10min post PHEA, a drop of SBP under 90mmHg occurred in 22/122(18%) of trauma, 26/69(38%) of post-OHCA and 14/49(28%) of medical cases, respectively (p=0.02). Dose reduction was demonstrated across three patient groups: no SBP<90mmHg, new drop to SBP<90mmHg within 10 minutes of PHEA, and pre-PHEA SBP<90mmHg, with median (IQR) doses of fentanyl/ ketamine of 2.1(1.7-2.9)mcg/kg / 1.4(1.0-2.0)mg/kg, 2.0(1.3-2.5)mcg/kg / 1.1(0.9-1.7)mg/kg, 1.0(0.6-1.0)mcg/kg / 0.9(0.6-1.0)mg/kg, respectively. Linear regression demonstrated neither higher doses of ketamine (p=0.57) or fentanyl (p=0.11) to be independently correlated with lower SBPs within 10min post PHEA.

**Conclusions**

Extending the post PHEA observation period in this study revealed a higher incidence of new hypotension using a fentanyl and ketamine induction protocol than previous major trauma studies^2,3,^. Dose reduction and linear regression analysis indicate that these results are not due to unrecognized instability or an inappropriate application of the published protocol. New post PHEA hypotension was significantly increased in OHCA and medical patients.

**References**

1. Stollings JL, Diedrich DA, Oyen LJ, et al.: Rapid-Sequence Intubation: A Review of the Process and Considerations When Choosing Medications. Annals of Pharmacotherapy. 2014;48(1):pp 62–76.

2. Lyon RM et al. Significant modification of traditional rapid sequence induction improves safety and effectiveness of pre-hospital trauma anaesthesia. Crit Care. 2015; 19(1): 134.

3. Newton A, Ratchford A and Khan, I. Incidence of adverse events during prehospital rapid sequence intubation: a review of one year on the London Helicopter Emergency Medical Service. J Trauma. 2008;64(2):pp 487-492.

## 57) The effectiveness of helicopter transport in the delivery of Pre Hospital Critical Care teams in a mixed rural and urban UK setting

### Lily Stanley^1^, Carla Swanson-Low^1^, Julian Thompson^1,2^, Jules Blackham^1,2^

#### ^1^Great Western Air Ambulance Charity, South West Ambulance Service Trust, Bristol UK; ^2^Severn Major Trauma Network, Bristol, UK

##### **Correspondence:** Lily Stanley (lilystanley04@gmail.com)

**Background**

Pre Hospital Critical Care (PHCC) teams^1^ provide advanced care to critically ill patients over wide geographical areas, frequently utilising helicopters as delivery vectors for time critical interventions. Although helicopters offer multiple roles in addition to team delivery^2^, some commentators have challenged the utility of helicopters in a UK setting^3^. This retrospective study analysed airbase dispatch to incident scene times of an air ambulance service using both air and land based transport to refine team deployment in future time-critical taskings.

**Methods**

A retrospective database analysis of Pre Hospital Emergency Anaesthesia (PHEA) cases undertaken by Great Western Air Ambulance Charity between September 2007 and June 2017.

**Results**

849 PHEA missions with complete data were identified; 54.4% (462/849) were attended by helicopter and 45.6%(387/849) by rapid response vehicle (RRV). When comparing transport from airbase to scene by helicopter vs RRV, mean distance was 20.5 vs 13.3 miles (p<0.001) and mean transport time 20.86 vs 18.1 minutes (p=0.001) respectively.

Average speed to scene was 60.3mph by helicopter and 48.7mph by RRV (p=0.0002). Linear trend analysis of time to scene by mode of transport demonstrates increasing time superiority of helicopter over RRV transport with increasing distance travelled.

Further analysis demonstrates that mean airbase to incident scene distances have increased over the study period from 15.2 miles in 2008 to 21.6 in 2017 (p=0.002), reflecting increased tasking to more remote cases. Patient transport to hospital data identified 16 receiving hospitals with transport distances of up to 98.5 miles from PHEA incident scene.

**Conclusions**

In addition to the cited accessory benefits of a helicopter transport vector^3^, this study demonstrates significantly increased speed to scene of helicopter versus RRV-deployed PHCC teams responding to time critical missions. This data provides support for the effective use of helicopters in the deployment of PHCC teams in a UK setting.

**References**

1. von Vopelius-Feldt J, Benger J Who does what in prehospital critical care? An analysis of competencies of paramedics, critical care paramedics and prehospital physicians Emerg Med J 2014;**31:**1009-1013.

2. Butler DP, Anwar I, Willett K Is it the H or the EMS in HEMS that has an impact on trauma patient mortality? A systematic review of the evidence Emergency Medicine Journal 2010;27:692-701.

3. Johnsen AS, Fattah S, Sollid SJM, et al Utilisation of helicopter emergency medical services in the early medical response to major incidents: a systematic literature review BMJ Open 2016;6:e010307.

## 58) Rib fractures: Implementing a Chest Injury Pathway in a district general hospital is associated with improvements in analgesia, standardization of care, and decreases in overall and pneumonia mortality

### Oliver Quick ^1^, Neil Roberts ^2^, Emma Harrison ^1^, Laura Shepherd ^1^, Rhys Owens ^1^, Ben Warrick ^1^

#### ^1^ Royal Cornwall Hospital, Truro, UK; ^2^ Derriford Hospital, Plymouth, UK

##### **Correspondence:** Oliver Quick (oli.quick@icloud.com)

**Background**

Rib fractures represent a significant proportion of trauma seen in Emergency Departments. Previous audit showed low rates of patient-controlled analgesia and regional anaesthesia, variable pathways of care and increased mortality compared to national average. A pathway was introduced to improve care.

**Methods**

Retrospective audit of all adult patients with rib/sternal fracture from trauma, admitted for active treatment to a district general hospital Aug 2017-Jan 2018. Patients were identified through TARN and WebPACS systems, imaging and notes were reviewed.

**Results**

70 patients identified for inclusion after review of 191 imaging reports and 80 sets of notes (increased from 43 previously). Overall 30-day mortality was 8.69%, reduced from 11.6%. 39 patients documented use of pathway (60%). 12 patients (18%) were treated for pneumonia, increased from 16%. Mortality was 0% in those treated for pneumonia, decreased from 29%. Median hospital LOS 7 days (range 1-37), increased from 6. Median ICU LOS was 4 days (range 1-7), increased from 3.

11 patients (17%) received regional anaesthesia, increased from 2 (5%) previously. 5 (8%) received erector spinae catheters. 7 patients (11%) received epidural anaesthesia (1 when ES catheter failed). 28 (44%) patients were prescribed PCAs, increased from 37% previously. 21 patients (33%) patients referred to cardiothoracics (increased from 23%).

Initial destination of care was CDU for 34 patients (53%), Critical Care for 15 (23%), 6 to MAU (9%), 6 to Respiratory (9%), 4 (6%) to other medical wards. 3 (out of 50) patients (6%) deteriorated on ward requiring escalation to ICU (9% previously).

**Conclusions**

Despite a large increase in number of patients, implementing a Chest Injury Pathway has been associated with increases in regional anaesthesia and patient-controlled analgesia, more patients getting a standardized pathway of care, less requiring secondary Critical Care admission, and decreases in mortality, particularly in pneumonia.

## 59) The demand for early advanced airway interventions in 70,550 UK trauma patients

### Kate Crewdson^1^, David Lockey^1,2^

#### ^1^North Bristol NHS Trust, Bristol, UK; ^2^Blizard Institute, Queen Mary University, London

##### **Correspondence:** Kate Crewdson (Kate.Crewdson@nbt.nhs.uk)

**Background**

Advanced airway management is a treatment priority in the early phase of trauma care. It is likely that a proportion of patients who receive urgent airway management in the Emergency Department (ED) represent an unmet demand for airway intervention in the pre-hospital phase of care. This study aimed to increase data on current UK emergency airway practice in major trauma patients and establish the degree of unmet demand that may still exist in this patient group.

**Method**

A retrospective review of the Trauma Audit & Research Network (TARN) database was performed to identify all patients admitted to UK Major Trauma Centres between 01/04/2012 and 27/06/2016. Airway interventions performed for each patient were included in the final analysis.

**Results**

Of 70,550 study patients, 11,010 patients (15.6%) underwent pre-hospital or ED airway interventions. 10,264 patients (93.2%) received urgent tracheal intubation; 4,375 (42.6%) in the pre-hospital setting and 5,889 in ED. 3,264 patients were intubated within 30 minutes of hospital arrival (75.4%). A further 1,593 patients had a pre-hospital non-intubation airway intervention, of whom 881 were intubated in ED, 91.4% within 30 minutes of arrival.

The mortality rate for patients requiring emergency intubation in any setting was 28.1%. 1,473 of 4,375 patients (33.7%) who required pre-hospital intubation died, and 1,511 patients (25.7%) died following ED intubation. 446 of 881 patients (50.6%) intubated in ED after receiving pre-hospital non-intubation interventions died, p<0.0001.

**Discussion**

Over 70% of ED intubations were carried out within 30 minutes of hospital arrival, which suggests that there may be significant unmet demand in pre-hospital advanced airway management for trauma patients. The significantly increased mortality of patients who had airway interventions but not pre-hospital anaesthesia compared with those that received a pre-hospital anaesthetic may indicate a benefit of pre-hospital anaesthesia and advanced airway management.

## 60) A Cross Sectional Analysis of Current Tranexamic Acid Use in US Trauma Centers

### Kham Ali^1^, Gentry Wilkerson^2^, Brandon R. Bruns^3^, Lucy S. Liu^4^

#### ^1^ Department of Emergency Medicine, St. John’s Riverside Hospital, Yonkers, NY, USA; ^2^ Department of Emergency Medicine, University of Maryland School of Medicine, Baltimore, MD, USA; ^3^ R Adams Cowley Shock Trauma Center, University of Maryland School of Medicine, Baltimore, MD, USA; ^4^ University of Maryland School of Medicine, Baltimore, MD, USA

##### **Correspondence:** Kham Ali (kali@gwmail.gwu.edu)

**Background**

The well-known CRASH-2 [1] and MATTERS [2] found a reduction of all-cause mortality with TXA usage but were potentially limited because of their generalizability. Regardless, trauma centers are using TXA outside of its FDA-approved indications. The purpose of this survey study was to understand current usage of TXA in trauma patients by trauma centers (TCs) within United States.

**Methods**

We performed a cross sectional survey recording the use of TXA at Level I and Level II TCs in the US. Trauma center managers and directors completed the surveys over a few weeks electronically from 4/2018-10/2018.

**Results**

95.9% of Level I and 89.6% of Level II TCs had TXA available regardless of indication. 59.8% of Level I and 75.3% of Level II TCs use TXA in MTP protocols. 63.9% of Level I and 66.2% of Level II TCs have protocols in place for TXA use in trauma. 60.82% of Level I and 28.57% of Level II TCs used viscoelastic testing to guide TXA usage. Of the institutions without a protocol, 18% considered a protocol, 27% never considered one, and 17% are developing one. The institutions that did not use TXA cited “lack of strong evidence” as a deterrent.

**Discussion**

Despite high use level of TXA, few TCs use any objective data (i.e. viscoelastic testing) for administration guidance and 1/3 of TXA users don’t have a formal protocol. Although it seems that better clinical use data and guidelines need to be elucidated, TCs seem unafraid to use TXA off-label.

**References**

1 CRASH-2 trial collaborators, Shakur H, Roberts I, et al. Effects of tranexamic acid on death, vascular occlusive events, and blood transfusion in trauma patients with significant haemorrhage (CRASH-2): a randomised, placebo-controlled trial. Lancet. 2010;376(9734):23-32. doi:10.1016/S0140-6736(10)60835-5.

2 Morrison JJ, Dubose JJ, Rasmussen TE, Midwinter MJ. Military Application of Tranexamic Acid in Trauma Emergency Resuscitation (MATTERs) Study. Arch Surg. 2012;147(2):113-119. doi:10.1001/archsurg.2011.287.

## 61) UK Adherence to NICE Guidance In The Operative Treatment Of Distal Radius Fractures – A Single Centre Experience

### Gregory Neal-Smith^†^, En Lin Goh^†^, Chris Bretherton

#### Department of Orthopaedic Surgery, Oxford University Hospitals, Oxford, UK

##### **Correspondence:** Gregory Neal-Smith (gregnealsmith@doctors.org.uk)

^†^Both authors contributed equally to this work

**Background**

The National Institute for Health and Care Excellence (NICE) guidance for the operative treatment of distal radius fractures recommend surgery within 72 hours of injury for intra-articular fractures and within 7 days for extra-articular fractures [1]. The DRAFFT Impact Study demonstrated that nationally, only 39% of intra-articular fractures were operated on within 72 hours and 60% of extra-articular fractures within 7 days. This audit aims to update on current adherence to NICE guidance [1].

**Methods**

All acute adult distal radius fractures managed operatively between September to November 2018 at the Horton General Hospital were included in this study. Data regarding gender, age, fracture classification (AO and Frykman classifications), procedure and time to surgery were retrospectively collected. Standards used in this audit were obtained from the NICE guideline NG38 [1].

**Results**

A total of 20 cases were identified. The mean time to operation was 4.5 days for intra-articular fractures and 4.9 days for extra-articular fractures. Additionally, 43% of intra-articular fractures were operated on within 72 hours and 69% of extra-articular fractures were operated on within 7 days.

**Discussion**

Both intra- and extra-articular fractures had a comparable delay to surgery of 4.5 and 4.9 days respectively. These were shorter than the delay to surgery reported in the DRAFFT Impact Study, which were 6.2 days for intra-articular fractures and 6.6 days for extra-articular fractures. Although the proportion of intra- and extra-articular fractures operated on within 72 hours and 7 days appear comparable with the national averages, there was a local improvement in the proportion of extra-articular fractures that were operated on within 7 days from 30% to 69%.

Time to surgery for intra-articular fractures remains unsatisfactory, with a significant proportion of these fractures failing to meet current standards. Streamlining the process for patients undergoing surgery will be required to improve this.

**Reference**

1. NICE Guidance NG38 - Fractures (Non-Complex): Assessment and Management [https://www.nice.org.uk/guidance/ng38]

